# Angiotensinogen in hepatocytes contributes to Western diet-induced liver steatosis[S]

**DOI:** 10.1194/jlr.M093252

**Published:** 2019-10-11

**Authors:** Xin-Ran Tao, Jia-Bing Rong, Hong S. Lu, Alan Daugherty, Peng Shi, Chang-Le Ke, Zhao-Cai Zhang, Yin-Chuan Xu, Jian-An Wang (王建安)

**Affiliations:** * Department of Cardiology, Cardiovascular Key Laboratory of Zhejiang Province, Second Affiliated Hospital, Zhejiang University School of Medicine, Hangzhou, China; † Saha Cardiovascular Research Center and Departments of Pharmacology and Nutritional Sciences and Physiology, University of Kentucky, Lexington, KY; § Second Affiliated Hospital, Zhejiang University School of Medicine, Hangzhou, China; ** Department of Intensive Care Unit, Second Affiliated Hospital, Zhejiang University School of Medicine, Hangzhou, China

**Keywords:** diet and diet lipids, triglycerides, fatty acid/biosynthesis, lipolysis and fatty acid metabolism, nuclear receptor/Srebp, nonalcoholic fatty liver disease

## Abstract

Nonalcoholic fatty liver disease (NAFLD) is considered as a liver manifestation of metabolic disorders. Previous studies indicate that the renin-angiotensin system (RAS) plays a complex role in NAFLD. As the only precursor of the RAS, decreased angiotensinogen (AGT) profoundly impacts RAS bioactivity. Here, we investigated the role of hepatocyte-derived AGT in liver steatosis. AGT floxed mice (hepAGT^+/+^) and hepatocyte-specific AGT-deficient mice (hepAGT^−/−^) were fed a Western diet and a normal laboratory diet for 12 weeks, respectively. Compared with hepAGT^+/+^ mice, Western diet-fed hepAGT^−/−^ mice gained less body weight with improved insulin sensitivity. The attenuated severity of liver steatosis in hepAGT^−/−^ mice was evidenced by histologic changes and reduced intrahepatic triglycerides. The abundance of SREBP1 and its downstream molecules, acetyl-CoA carboxylase and FASN, was suppressed in hepAGT^−/−^ mice. Furthermore, serum derived from hepAGT^+/+^ mice stimulated hepatocyte SREBP1 expression, which could be diminished by protein kinase B (Akt)/mammalian target of rapamycin (mTOR) inhibition in vitro. Administration of losartan did not affect diet-induced body weight gain, liver steatosis severity, and hepatic p-Akt, p-mTOR, and SREBP1 protein abundance in hepAGT^+/+^ mice. These data suggest that attenuation of Western diet-induced liver steatosis in hepAGT^−/−^ mice is associated with the alternation of the Akt/mTOR/SREBP-1c pathway.

Nonalcoholic fatty liver disease (NAFLD) is defined as excessive liver lipid accumulation (triglyceride content more than 5% of liver weight) excluding other competing causes of hepatic steatosis (1). With a prevalence among adults reaching about 25%, NAFLD is now the most common liver disease worldwide (2). Highly accompanied by obesity and dyslipidemia, NAFLD is recognized as a typical liver manifestation of metabolic syndrome, which may arise from insulin resistance (3). Additionally, NAFLD is also considered as a major contributor to cardiovascular disease and obesity-related comorbidities (4, 5).

Previous studies have shed a light on the effect of the renin-angiotensin system (RAS) on NAFLD. It has been suggested that inhibition or deletion of critical components of the RAS, including renin, angiotensin converting enzyme, or angiotensin type 1 receptor (AT1R), may protect rodents from diet-induced liver steatosis (6–9). However, results from previous studies have been equivocal. For instance, Verbeek et al. (10) reported that losartan, an angiotensin receptor blocker, failed to improve NAFLD in obese mice. It was also suggested that telmisartan, another angiotensin receptor blocker, significantly ameliorated liver triglyceride accumulation via partial PPARγ agonism, rather than RAS inhibition (11). This controversial evidence has implicated the complexity of the RAS as well as the interaction among its components; thus, manipulating one certain component of the RAS may trigger diverse changes. Moreover, unexpected side effects of RAS intervention would interfere with the outcomes.

Angiotensinogen (AGT) is a polypeptide consisting of 485 amino acid residues (12). It can be cleaved to generate angiotensin I, angiotensin II (Ang II), and other angiotensin peptides of the RAS (13). AGT is expressed in multiple organs including brain, kidney, and adipose tissues; however, AGT derived from hepatocytes alone contributes up to 90% of circulating AGT (14). As a unique substrate of the RAS, reduction of AGT profoundly impacts RAS activation. Therefore, it is reasonable to evaluate the overall impact of AGT deletion on the influence of the RAS on NAFLD. A previous study has revealed that high-fat diet-fed global AGT KO mice showed reduced body weight and less adipose tissue (15). However, whether AGT deficiency affects diet-induced liver steatosis has not been reported. Recently, Lu et al. (14) have reported that both pharmaceutical and genetic repression of hepatocyte-specific AGT protected mice from Western diet-induced liver triglyceride deposition. Yet, the underlying mechanism remains unclear.

Hence, the current study was designed to figure out the role of the RAS in development of NAFLD using a hepatocyte-specific AGT-deficient (hepAGT^−/−^) mouse model. By comparing hepatic changes between hepAGT^+/+^ and hepAGT^−/−^ mice, we aimed to elucidate the impact of hepatocyte-derived AGT on NAFLD and its potential mechanisms.

## MATERIALS AND METHODS

### Animals and diet

AGT floxed mice with and without transgenic Alb-Cre of C57BL/6 background were kindly provided by Dr. Alan Daugherty’s laboratory in Saha Cardiovascular Research Center, University of Kentucky. The hepAGT^−/−^ mice were generated by crossing Alb-Cre^+/−^ AGT floxed mice (male) to AGT floxed mice (female) as described previously (14). As expected, the mRNA and protein abundance of AGT were significantly decreased in hepAGT^−/−^ mice compared with hepAGT^+/+^ littermates (supplemental Fig. S1).

In the current study, all mice were maintained in a barrier facility on a light:dark cycle of 12:12 h (ambient temperature of 23°C) and fed a normal mouse laboratory diet. At the age of 8–10 weeks, male hepAGT^−/−^ and hepAGT^+/+^ mice were divided into either the normal laboratory diet group or the Western diet group. Western diet is defined as a diet enriched with saturated fat (42% calories from fat, Diet #TD.88137; Harlan Teklad). Body weight and food consumption were checked weekly. All animal studies were approved by the Animal Care and Use Committee of Zhejiang University in accordance with the Chinese guidelines for the care and use of laboratory animals.

### Intraperitoneal glucose tolerance test and insulin tolerance test

An intraperitoneal glucose tolerance test (IPGTT) and an insulin tolerance test (ITT) were performed as described (16). Briefly, for the IPGTT, mice were fasted overnight (16 h) and then fasting blood glucose concentration measurement was performed. Next, normal saline containing 20% (w/v) glucose (2 g glucose per kilogram body weight) was intraperitoneally injected into each mouse, and blood samples were collected at 15, 30, 60, and 120 min after injection for further blood glucose measurement. For the ITT, after a 5 h fast, the fasting blood glucose concentration of each individual mouse was measured. Insulin (Novolin R; Novo Nordisk, Bagsvaerd, Denmark) was then injected intraperitoneally (0.75 unit of insulin per kilogram body weight). The concentration of blood glucose was measured at 15, 30, 60, and 120 min after insulin injection. The IPGTT and ITT results were analyzed as the incremental area under the blood glucose curve (for IPGTT) and area under the curve of percent change from baseline glucose (for ITT) for interpretation of insulin sensitivity, respectively.

### Plasma insulin, alanine transaminase, and aspartate transaminase concentration measurement

Mouse blood samples were collected via right ventricle with EDTA (1.8 mg/ml) after 5 h fasting and then centrifuged at 400 *g* at 4°C for 20 min to separate plasma. Plasma insulin concentration was measured by insulin ELISA kit (10-1247-01; Mercodia, Uppsala, Sweden) following the manufacturer’s instructions. Plasma triglyceride concentration was measured using a triglyceride measurement kit (E1003; Applygen Technologies Inc., Beijing, China). Plasma alanine transaminase (ALT) and aspartate transaminase (AST) activity was detected by ALT assay kit (C009-2; Njjcbio, Jiangsu, China) and AST assay kit (C010-2; Njjcbio), respectively.

### The measurement of liver tissue triglyceride, free cholesterol, and cholesteryl ester concentration

Liver tissues were snap-frozen in liquid nitrogen at euthanization, placed in a −80°C refrigerator, and analysis was performed within a month. The liver tissue samples were homogenized by tissue lysis buffer using tissue triglyceride or cholesterol assay kits (E1013 for tissue triglyceride assay, E1015 for tissue free cholesterol assay, and E1016 for tissue total cholesterol assay; Applygen Technologies Inc.), and the measurement of liver tissue triglyceride, free cholesterol, and cholesteryl ester concentrations was further carried out following the manufacturer’s manuals. The concentration of tissue cholesteryl ester was calculated as follows: Concentration (cholesteryl ester) = Concentration (total cholesterol) – Concentration (free cholesterol).

### Quantitative real-time PCR

Total mRNA was extracted from liver tissue by TRIzol (TRIzol reagent; Invitrogen) and reverse-transcribed to cDNA using PrimeScript™ RT Master Mix (Perfect Real Time) (RR036A; Takara, Beijing, China). The sequences of primers utilized in the current study are listed in **Table 1**. Quantitative real-time PCR (qPCR) assays were performed using One Step TB Green™ PrimeScript™ RT-PCR kit (Perfect Real Time) (RR066A; Takara). The mRNA abundance of target genes was normalized to β-actin and analyzed using the ΔΔCt method for quantification.

**TABLE 1.** Primer sequences for qPCR

Gene	Forward Primer (5′→3′)	Reverse Primer (5′→3′)
m*Agt*	GTACAGACAGCACCCTACTT	CACGTCACGGAGAAGTTGTT
m*β-actin*	AGATCAAGATCATTGCTCCTCCT	ACGCAGCTCAGTAACAGTCC
m*Srebf1*	GGGCAAGTACACAGGAGGAC	AGATCTCTGCCAGTGTTGCC
m*Fasn*	AAGCAGGCACACACAATGGA	AGTGTTCGTTCCTCGGAGTG
m*Acc*	GCGATACACTCTGGTGCTCA	CCCAGGGAAACCAGGATATT
m*Scd1*	CATGCGATCTATCCGTCGGT	CCTCCAGGCACTGGAACATAG
m*Dgat2*	AGTGGCAATGCTATCATCATCGT	AAGGAATAAGTGGGAACCAGA
m*Lxr*	GCTGCTTCGTGACCCACTAT	CTGTCTCCCCATCTCACCCA
m*Cd36*	GCCAAGCTATTGCGACATGAT	CAGATCCGAACACAGCGTAGA
m*Ucp2*	TCTGCACTCCTGTGTTCTCCT	TAGAAAATGGCTGGGAGACGA
m*Slc27a2*	ATCGTGGTTGGGGCTACTTTAG	TTTGGTTTCTGCGGTGTGTTG
m*Slc27a5*	GAGGGCAATGTGGGCTTAATG	AGGCTCTGCTGTCTCTATGTC
m*Cide-a*	TGACATTCATGGGATTGCAGAC	GGCCAGTTGTGATGACTAAGAC
m*Cide-b*	GCTGCTACGTGGAGTGCTAA	ACAACATCCCACTCTTGGGG
m*Cide-c*	ATGGACTACGCCATGAAGTCT	CGGTGCTAACACGACAGGG
m*Pparγ*	TCCTCATCTCAGAGGGCCAA	ATGTCCTCGATGGGCTTCAC
m*Lipc*	CATTTTCCTGGTGTTCTGCATCT	TAGCAAGCCATCCACCGAC
m*Mgll*	TGGAAAAGTGGCGACATGAG	TCTTTAGGCCCTGTTTCCATT
m*Pparα*	CACGATGCTGTCCTCCTTGAT	GCCAGGCCGATCTCCA
m*Fgf21*	AGCATACCCCATCCCTGACT	AGGAGACTTTCTGGACTGCG
m*Acox1*	ACCTTCCCTTTCTTGCTTTGC	GCTTTCCTGTGATTTC.TGGTGT
m*Cpt1a*	ATGGACCCCACAACAACGG	TCATCAGCAACCGGCCCAAA
m*Acadl*	CCGATTGCCAGCTAATGCCT	TGCTTCACGTAGTTCCTGGT
m*PPARgc1a*	CGCCGTGTGATTTACGTTGG	GCTGTCTCCATCATCCCGC

### Western blotting

Total protein from hepatocytes and liver were extracted using RIPA lysis buffer (P0013B; Beyotime, Shanghai, China). The proteins were separated by SDS-PAGE gel, then transferred to PVDF membranes (Millipore, Darmstadt, Germany), and immunoblotted by primary antibodies according to the manufacturers’ manuals. The primary antibodies were detected by a HRP-conjugated secondary antibody from the appropriate species and reacted with Immobilon Western Chemiluminescent HRP Substrate (Millipore). Primary antibodies against the following proteins were used: AGT (IBL-America #JP28101, RRID: AB_2341481), SREBP1 (Abcam #ab28481, RRID: AB_778069), acetyl-CoA carboxylase (ACC) (Cell Signaling Technology #3676, RRID: AB_2219397), FASN (Abcam #ab128856, RRID: AB_11143234), phospho-protein kinase B (Akt) (Ser473) (Cell Signaling Technology #4060, RRID: AB_2315049), Akt (pan) (Cell Signaling Technology #4691, RRID: AB_915783), β-actin (KC-5A08, Aksomics Inc., Shanghai, China), cluster of differentiation 36 (CD36) (Abcam #ab133625, RRID: AB_2716564), LC3A/B (Cell Signaling Technology #4108, RRID: AB_2137703), P62 (Cell Signaling Technology #5114, RRID: AB_10624872), ATG7 (Cell Signaling Technology #8558, RRID: AB_10831194), and ATG12 (Cell Signaling Technology #4180, RRID: AB_1903898).

### Histology

Livers were dissociated and then embedded in proper embedding medium. Paraffin-embedded sections at 3–4 μm thickness were stained with H&E. In addition, liver tissue crystal sections embedded with Optimal Cutting Temperature Compound (4583; Sakura) were stained with Oil Red O. The images were visualized by phase-contrast light microscopy.

### Autophagic flux assays

Mice were fasted for 5 h and then treated with bafilomycin A1 (S1413; Selleck Chemicals; 2.5 mg/kg body weight) or vehicle (DMSO, D2650; Sigma, Saint Louis, MO) via intraperitoneal injection. Three and one-half hours after injection, the mice were euthanized and liver microtubule-associated protein 1 light chain 3 (LC3)-II protein abundance was quantified using Western blotting for assessment of hepatic autophagic flux as previously described (17).

### The isolation of liver mitochondria

Mitochondria in liver were isolated using a tissue mitochondria isolation kit (C3606; Beyotime). Briefly, 100 mg of fresh liver tissue were collected, washed, and minced in ice-cold PBS. Then the tissue pieces were transferred to a precooled glass homogenizer with 1 ml of mitochondria isolation buffer and homogenized with 10 strokes at medium speed, and then centrifuged at 600 *g* at 4°C for 5 min. The supernatant was then transferred to a new tube and centrifuged at 3,500 *g* at 4°C for 10 min. Then the supernatant was carefully transferred to a new tube for cytoplasmic protein quantitation. The remaining mitochondrial pellets were washed with 1 ml of isolation buffer and resuspended in 100 μl of mitochondrial preserving solution on ice for further analysis.

### Evaluation of liver mitochondrial fatty acid oxidation

The fatty acid oxidation capacity of isolated liver mitochondria was measured using an Oxygraph-2k machine (O2k; OROBOROS Instruments, Innsbruck, Austria). Briefly, the isolated liver mitochondria in 100 μl of preserving solution were added to the chamber with 2 ml of MiR05 [respiration media containing 0.5 mM EGTA, 3 mM MgCl_2_·6H_2_O, 60 mM potassium lactobionate, 20 mM taurine, 10 mM KH_2_PO_4_, 20 mM HEPES, 110 mM sucrose, and 1 g/l fatty acid-free BSA (pH 7.1)]. Malate (0.25 mM), palmitoylcarnitine (2.5 μM) (P4509; Sigma), octanoylcarnitine (0.2 mM) (0605; TOCRIS Bioscience), and ADP+Mg^2+^ (1.25 mM) were used as substrates for measuring β-oxidation. The capacity of mitochondrial fatty acid oxidation was interpreted by the maximal oxygen consumption rate, which was calculated as the difference of oxygen flux between pre- and post-treatment with 0.5 μM titration of rotenone (45656; Sigma).

### Administration of losartan in hepAGT^+/+^ mice

Losartan was continuously administered to mice for 12 weeks. Briefly, Model 2006 Alzet mini-osmotic pumps (Durect Corporation) were implanted subcutaneously into male hepAGT^+/+^ mice aged 8–10 weeks to deliver either 0.9% saline (vehicle) or losartan (61188; Sigma; 10 mg/kg/day). The osmotic pumps were equipped following the manufacturer’s instructions. Pumps were replaced every 6 weeks post first implantation.

### Primary hepatocyte isolation, culture, and treatment

The isolation and culture of primary hepatocytes were performed as described (18, 19). Briefly, under anesthesia, the hepatic portal vein and inferior vena cava of each mouse were fully exposed. The liver was sufficiently perfused with D-Hank’s balanced salt solution via the hepatic portal vein. The liver was next perfused with 50 ml of prewarmed digestive solution containing 0.85 mg/ml type II collagenase (17101015; Gibco). Then, the whole liver was taken down and rinsed in DMEM containing 10% FBS in a sterile dish. Hepatocytes were liberated by tearing the liver capsule. The suspension was filtered by sterile 100 μm strainer and centrifuged at 50 *g* at 4°C for 2 min. The supernatant was carefully discarded and the cell pellets were resuspended with DMEM containing 10% FBS and centrifuged at 50 *g* at 4°C for 2 min. After repeating this step one more time, hepatocyte pellets were then resuspended with DMEM containing 10% FBS and plated to culture plates precoated with rat tail collagen. The plating medium was replaced with serum-free DMEM 5 h after plating, and hepatocytes were cultured overnight before subsequent experiments. All experiments were finished within 48 h after plating.

Primary hepatocytes were incubated with DMEM containing 0.5 mM palmitic acid and 10 nM insulin (Novolin R; Novo Nordisk) to induce steatosis. In the serum stimulation experiments, the above medium was added with 10% serum from hepAGT^+/+^ and hepAGT^−/−^ mice, respectively. Serum used in this experiment was obtained from hepAGT^+/+^ and hepAGT^−/−^ mice fed a normal laboratory diet after 5 h of fasting. For Akt and mammalian target of rapamycin (mTOR) signaling inhibition, primary hepatocytes were pretreated with 10 μM of MK2206 dihydrochloride (MK2206) (HY-10358; MCE) for 2 h and 20 nM of rapamycin (S1039; Selleck Chemicals) for 30 min to inhibit Akt and mTOR activity, respectively.

### Statistical analysis

All quantitative normal data were presented as mean ± SD. Comparison between groups was performed via Student’s *t*-test (comparison between two groups), one-way ANOVA or two-way ANOVA (comparison for more than two groups) for data that passed the normality test. Data that did not pass the normality test were expressed as median (minimum, maximum) and analyzed by ANOVA on ranks. Statistical analyses were completed using Sigma Plot 12.0 software. *P*-values less than 0.05 were considered statistically significant.

## RESULTS

### HepAGT^−/−^ mice resistant to Western diet-induced body weight gain and insulin resistance

**Fig. 1.**
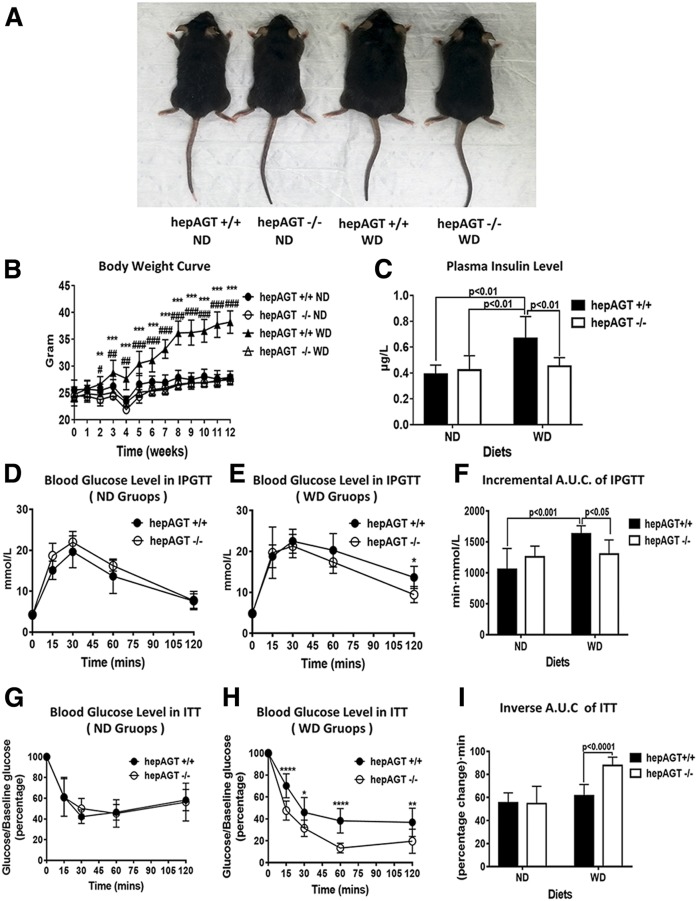
HepAGT^−/−^ mice resistant to Western diet-induced body weight gain with improved insulin sensitivity. A: Representative image of hepAGT^+/+^ and hepAGT^−/−^ mice fed a normal laboratory diet and Western diet, respectively, for 12 weeks. B: Hepatocyte-specific AGT deficiency showed modest effect on mouse body weight growth when fed a normal laboratory diet, whereas hepAGT^−/−^ mice gained less body weight than hepAGT^+/+^ mice in response to Western diet (N = 7–9 for each group). **P < 0.01, ***P < 0.001 versus hepAGT^−/−^ WD; #*P* < 0.05, ##*P* < 0.01, ###*P* < 0.001 versus hepAGT^+/+^ ND. Comparison among groups by two-way ANOVA, S-N-K post hoc test. C: The concentration of fasting plasma insulin was similar between normal laboratory diet-fed hepAGT^+/+^ and hepAGT^−/−^ mice. However, an elevation of plasma insulin level was induced by Western diet in hepAGT^+/+^ mice, which could be attenuated after depletion of hepatic AGT (N = 5–10 for each group). Comparison among groups by two-way ANOVA, S-N-K post hoc test. D, G: Blood glucose levels measured in IPGTT (D) and ITT (G) were not different between hepAGT^−/−^ and hepAGT^+/+^ mice fed a normal laboratory diet (N = 6–8 for each group). Comparison between genotypes by Student’s *t*-test. E, H: Blood glucose levels measured in IPGTT (E) and ITT (H) were lower in hepAGT^−/−^ mice than those of hepAGT^+/+^ mice after 12 weeks of Western diet feeding (N = 8–9 for each group). *P < 0.05, **P < 0.01 versus hepAGT^−/−^. Comparison between genotypes by Student’s *t*-test. F: For IPGTT, incremental area under the glucose curve was significantly smaller in the Western diet-fed hepAGT^−/−^ mice than that of hepAGT^+/+^ littermates (ND: 1,054 ± 337 for hepAGT^+/+^ vs. 1,255 ± 176 for hepAGT^−/−^; WD: 1,629 ± 129 for hepAGT^+/+^ vs. 1,299 ± 2,332 for hepAGT^−/−^, *P* < 0.01; N = 6–9 for each group). Comparison among groups by two-way ANOVA, S-N-K post hoc test. I: For ITT, inverse area under the glucose curve was significantly elevated in Western diet-fed hepAGT^−/−^ mice than that of hepAGT^+/+^ littermates (ND: 55 ± 9 for hepAGT^+/+^ vs. 55 ± 15 for hepAGT^−/−^; WD: 61 ± 10 for hepAGT^+/+^ vs. 88 ± 7 for hepAGT^−/−^, *P* < 0.001; N = 6–9 for each group). Comparison among groups by two-way ANOVA, S-N-K post hoc test. ND, normal laboratory diet; WD, Western diet; A.U.C., area under the curve.

Eight- to ten-week-old male hepAGT^−/−^ and hepAGT^+/+^ mice were fed a normal laboratory diet or Western diet, respectively, for 12 weeks. Body weight and food intake were measured weekly. No difference in body weight was observed among all groups at baseline when fed a normal laboratory diet. However, after 3 weeks of Western diet feeding, hepAGT^−/−^ mice started to exhibit significantly less body weight gain than hepAGT^+/+^ mice (25.1 ± 1.3 g for hepAGT^−/−^ vs. 28.8 ± 2.3 g for hepAGT^+/+^, *P* < 0.01). At the end of the 12 week Western diet feeding, the body weight gain of hepAGT^−/−^ mice was significantly lower than that of hepAGT^+/+^ counterparts (27.9 ± 0.2 g for hepAGT^−/−^ vs. 38.2 ± 0.7 g for hepAGT^+/+^, *P* < 0.001) (**Fig. 1A**, B). In addition, hepAGT^−/−^ mice also displayed reduction in Western diet-induced adipose tissue increase (supplemental Fig. S2G–M). However, total food consumption was not affected by deficiency of hepatocyte-specific AGT either in normal diet or Western diet cohorts (supplemental Fig. S2A, B). These data support that loss of hepatocyte-derived AGT prevents Western diet-induced obesity.

Obesity is usually associated with insulin resistance, which serves as a significant characteristic of metabolic disorders. Therefore, the plasma insulin concentration of hepAGT^+/+^ and hepAGT^−/−^ mice was measured. Western diet induced an elevation of plasma insulin in hepAGT^+/+^ mice, which could be attenuated by deletion of hepatic AGT (Fig. 1C). We then performed IPGTT and ITT to assess systemic glucose tolerance and insulin sensitivity, respectively. When fed a normal laboratory diet, the blood glucose level was not different between hepAGT^−/−^ and hepAGT^+/+^ mice in both IPGTT and ITT tests (Fig. 1D, G; supplemental Fig. S2C–F). In cohorts of mice fed a Western diet, the incremental area under the curve of the blood glucose curve in the IPGTT was reduced, and the inverse area under the curve of the blood glucose curve in the ITT was increased in the hepAGT^−/−^ group compared with the hepAGT^+/+^ group (Fig. 1E, F, H, I; supplemental Fig. S2C–F), which implicates that glucose tolerance and insulin sensitivity were improved in hepAGT^−/−^ mice. Collectively, all these data reveal that hepatocyte-derived AGT deficiency prevents Western diet-induced body weight gain with improved insulin sensitivity.

### HepAGT^−/−^ mice were protected from Western diet-induced liver steatosis

**Fig. 2.**
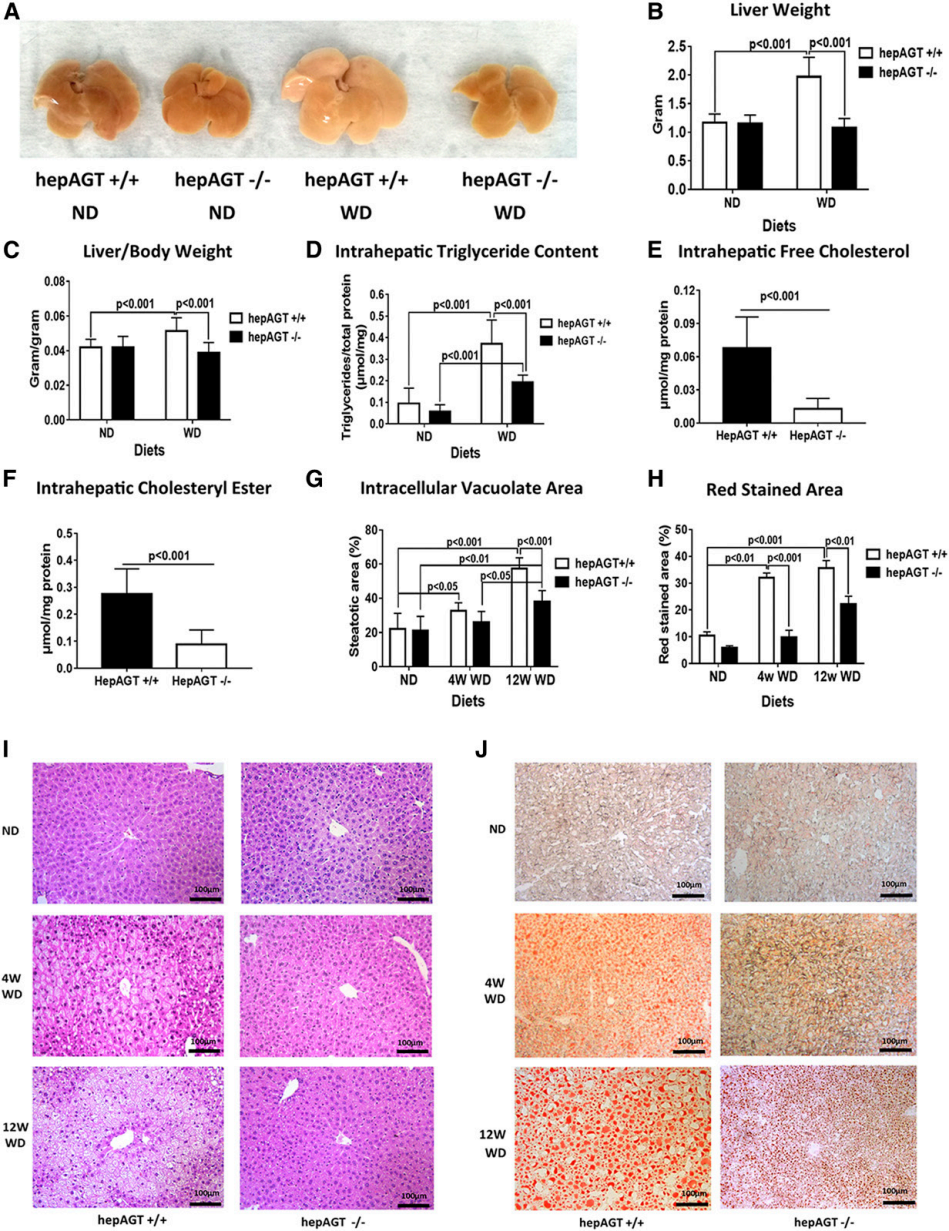
Hepatocyte-specific AGT deficiency attenuated Western diet-induced liver steatosis. A: Representative image of liver tissues from hepAGT^−/−^ and hepAGT^+/+^ mice fed a normal laboratory diet and Western diet, respectively, for 12 weeks. B: The reduction of liver weight was observed in hepAGT^−/−^ mice compared with hepAGT^+/+^ mice after 12 weeks of Western diet feeding. (2.0 ± 0.3 g for hepAGT^+/+^ vs. 1.1 ± 0.2 g for hepAGT^−/−^, *P* < 0.001; N = 7–9 for each group). Comparison among groups by two-way ANOVA, S-N-K post hoc test. C: The calculated liver/body weight ratio was decreased in the Western diet-fed hepAGT^−/−^ group versus the hepAGT^+/+^ group (0.05 ± 0.01 for hepAGT^+/+^ vs. 0.04 ± 0.01 for hepAGT^−/−^, *P* < 0.001; N = 7–9 for each group). Comparison among groups by two-way ANOVA, S-N-K post hoc test. D: Compared with hepAGT^+/+^ mice, intrahepatic triglyceride contents were remarkably lower in hepAGT^−/−^ mice fed a Western diet for 12 weeks (ND: 0.057 ± 0.032 μmol/mg for hepAGT^−/−^ vs. 0.095 ± 0.071 μmol/mg for hepAGT^+/+^; WD: 0.096 ± 0.032 μmol/mg for hepAGT^−/−^ vs. 0.37 ± 0.11 μmol/mg for hepAGT^+/+^, *P* < 0.001; N = 7–8 for each group). Comparison among groups by two-way ANOVA, S-N-K post hoc test. E: Western diet-fed hepAGT^−/−^ mice exhibited a reduction of intrahepatic free cholesterol contents compared with hepAGT^+/+^ mice. (0.013 ± 0.009 μmol/mg for hepAGT^−/−^ vs. 0.068 ± 0.028 μmol/mg for hepAGT^+/+^, *P* < 0.001; N = 8–9 for each group). Comparison between genotypes by Student’s *t*-test. F: Western diet-fed hepAGT^−/−^ mice exhibited a reduction of intrahepatic cholesteryl ester contents compared with hepAGT^+/+^ mice (0.088 ± 0.054 μmol/mg for hepAGT^−/−^ vs. 0.275 ± 0.094 μmol/mg for hepAGT^+/+^, *P* < 0.001; N = 8–9 for each group). Comparison between genotypes by Student’s *t*-test. G: Quantification of the percentage of intracellular vacuolation area in liver tissue section stained with H&E staining (N = 4–6 for each group). Comparison among groups by two-way ANOVA, S-N-K post hoc test. H: Quantification of the percentage of red-stained area in liver tissue section with Oil Red O staining (N = 4–6 for each group). Comparison among groups by two-way ANOVA, S-N-K post hoc test. I: The typical images of liver tissue sections stained with H&E (Scale bar = 100 μm). J: The typical images of liver tissue sections stained with Oil Red O staining (Scale bar = 100 μm). ND, normal laboratory diet; WD, Western diet.

Liver steatosis is a typical hepatic manifestation of metabolic disorders; therefore, we next evaluated the effect of hepatocyte-specific AGT deficiency on Western diet-induced liver steatosis. Twelve weeks of Western diet feeding induced a remarkable liver enlargement in hepAGT^+/+^ mice relative to hepAGT^−/−^ mice (**Fig. 2A**). Accordingly, the liver weight as well as liver-body weight ratio were significantly lower in hepAGT^−/−^ mice than in hepAGT^+/+^ mice (Fig. 2B, C; supplemental Fig. S3A).

The prominent feature of liver steatosis is excessive lipid accumulation in liver tissue. H&E and Oil Red O staining displayed obvious reductions of hepatocyte vacuolation and lipid-loading in liver tissues of hepAGT^−/−^ mice as compared with those of hepAGT^+/+^ mice when fed a Western diet (Fig. 2G–J). Next, the contents of lipid deposits in hepatic tissues were quantified. Consistent with the histological changes, the contents of the intrahepatic lipid profile including triglycerides, free cholesterol, and cholesteryl ester were remarkably lower in Western diet-fed hepAGT^−/−^ mice versus the hepAGT^+/+^ counterparts (Fig. 2D–F; supplemental Fig. S3B).

Liver steatosis can induce hepatic injury. Thus, we further measured the activity of plasma ALT and AST. Western diet increased the plasma ALT concentrations in hepAGT^+/+^ mice but not in hepAGT^−/−^ mice (supplemental Fig. S3C). However, plasma AST concentrations were not different between hepAGT^−/−^ and hepAGT^+/+^ mice (supplemental Fig. S3D). Collectively, our results indicate that hepatocyte-specific AGT deficiency attenuates Western diet-induced liver steatosis.

### Hepatocyte-specific AGT deficiency exhibited minor effects on liver fatty acid utilization

**Fig. 3.**
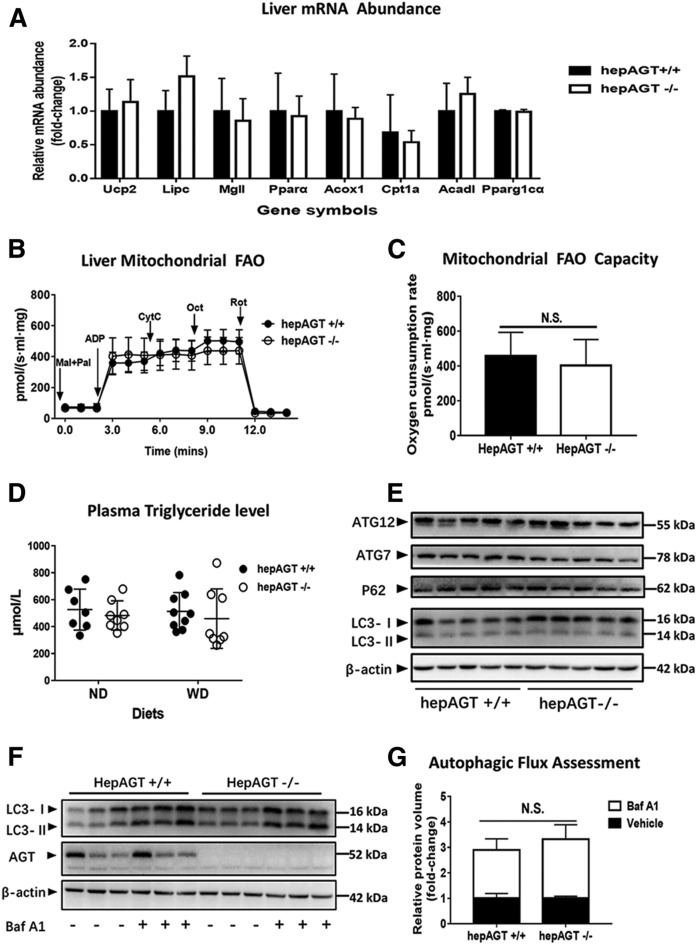
Deficiency of AGT in hepatocytes had minor effects on hepatic lipid utilization. A: Liver mRNA abundances of major genes involved in lipolysis, thermogenesis, and fatty acid oxidation were not different between hepAGT^−/−^ and hepAGT^+/+^ mice fed a Western diet (N = 5–9 for each group). Comparison between genotypes by Student’s *t*-test. B: The fatty acid oxidation assays of liver mitochondria isolated from hepAGT^+/+^ mice or hepAGT^−/−^ mice fed a Western diet, respectively (N = 3 for each group). C: The capacity of liver mitochondria fatty acid oxidation interpreted by oxygen consumption rate was similar between hepAGT^−/−^ mice and hepAGT^+/+^ mice fed a Western diet using a high-resolution respirometry (N = 3 for each group). Comparison between genotypes by Student’s *t*-test. D: The contents of plasma triglycerides were similar irrespective of genotype and diet (N = 6–9 for each group). Comparison among groups by ANOVA on Ranks. E: The abundance of autophagy-related proteins in liver tissues from hepAGT^−/−^ and hepAGT^+/+^ mice fed a Western diet (N = 5 for each group). F: The protein abundance of liver LC3 was detected in hepAGT^+/+^ and hepAGT^−/−^ mice fed a Western diet receiving either Baf A1 or vehicle, respectively (N = 3 for each group). G: Autophagic flux index calculated as the ratio of LC3-II protein abundance post/pre-Baf A1 injection was similar between hepAGT^+/+^ and hepAGT^−/−^ mice fed a Western diet (N = 3 for each group). Comparison between genotypes by Student’s *t*-test. ND, normal laboratory diet; WD, Western diet; FAO, fatty acid oxidation; Mal, malate; Pal, palmitoylcarnitine; Oct, octanoylcarnitine; CytC, cytochrome C; ADP, adenosine diphosphate; Rot, rotenone; ATG7, autophagy related 7; ATG12, autophagy related 12; P62, also known as SQSTM1; Baf A1, bafilomycin A1; N.S., not significant.

Hepatic triglyceride accumulation usually results from the imbalance between lipid import and consumption. A decrease in lipid export, fatty acid oxidation, lipolysis, and/or even autophagy can result in impaired lipid expenditure. To better understand the mechanisms by which hepatic AGT deletion influenced hepatic steatosis, liver mRNA expression of critical genes involved in the above-mentioned bio-processes was quantified by qPCR. No statistical differences in the expression of specific genes related to thermogenesis (*Ucp2*), lipolysis (*Lipc*), and fatty acid oxidation (*Mgll*, *Pparα*, *Acox1*, *Cpt1a*, *Acadl*, and *Pparg1cα*) were identified between hepAGT^+/+^ and hepAGT^−/−^ mice irrespective of diet (**Fig. 3A**, supplemental Fig. S4A).

Mitochondria are the pivotal organelles for energy metabolism in hepatocytes; therefore, we compared respiratory function of hepatic mitochondria from both hepAGT^+/+^ mice and hepAGT^−/−^ mice. Neither hepatocyte mitochondrial fatty acid oxidation capacity (Fig. 3B, C) nor total respiratory function (supplemental Fig. S4B, C) was affected by hepatic AGT deletion, suggesting that hepatocyte-derived AGT may not contribute to mitochondrial lipid utilization.

Lipids are mainly exported from liver via lipoprotein secretion, which contributes to plasma triglycerides. However, the levels of plasma triglycerides in all groups were not different regardless of genotype or diet treatment, indicating that hepatocyte-derived AGT might not affect lipoprotein secretion. (Fig. 3D).

Autophagy is a conserved quality-control process that participates in lipid droplet degradation, whose dysfunction contributes to the pathophysiology of NAFLD. Therefore, to verify whether liver autophagy is affected by hepatocyte-specific AGT deletion, the abundance of autophagy-related proteins was detected. However, a difference in protein abundance of hepatic P62, LC3-II, ATG7, and ATG12 was not identified between hepAGT^+/+^ mice and hepAGT^−/−^ mice (Fig. 3E). In addition, to further investigate the dynamics of hepatic autophagy, hepAGT^+/+^ mice and hepAGT^−/−^ mice were treated with bafilomycin A1, an autophagy-blocking agent. The results support that hepatic AGT deficiency does not affect hepatic autophagic flux (Fig. 3F, G). In summary, these findings indicate that hepatic AGT exerts minor effects on liver fatty acid utilization.

### Hepatocyte-specific AGT deficiency was associated with a remarkable suppression on the hepatic SREBP-1c pathway

Given that the above findings suggested that the absence of hepatocyte-derived AGT exerted minimal effects on liver fatty acid utilization, we further investigated to determine whether hepatic AGT deficiency affected pathways involved in lipid importation, including fatty acid uptake, lipid biosynthesis, and lipid storage.

CD36 and fatty acid transport proteins (FATPs) are major contributors to fatty acid uptake in liver and are involved in the development and progression of NAFLD (20). Among the isoforms of FATPs, FATP2 and FATP5, encoded by *Slc27a2* and *Slc27a5*, respectively, are predominantly expressed in the liver. However, liver mRNA expression of *Slc27a2* and *Slc27a5* was not different between hepAGT^+/+^ and hepAGT^−/−^ mice regardless of diet (supplemental Fig. S5A, B). Also, a difference of mRNA and protein abundance of CD36 between hepAGT^+/+^ and hepAGT^−/−^ mice was not detected (supplemental Fig. S5C, D). The findings above indicate that hepatic AGT may not be involved in the process of fatty acid uptake.

De novo lipogenesis and esterification are the two major pathways contributing to hepatic lipid synthesis. De novo lipogenesis is defined as fatty acid synthesis from acetyl-CoA, while esterification refers to the conversion of free fatty acid to triglyceride with glycerol (4). As shown by qPCR assays, hepatic mRNA abundance of diacylglycerol acyltransferase 2 (*Dgat2*), a key enzyme of esterification, was not affected by hepatocyte AGT deficiency, suggesting that hepatocyte-derived AGT may not participate in the process of esterification (supplemental Fig. S5A, B).

**Fig. 4.**
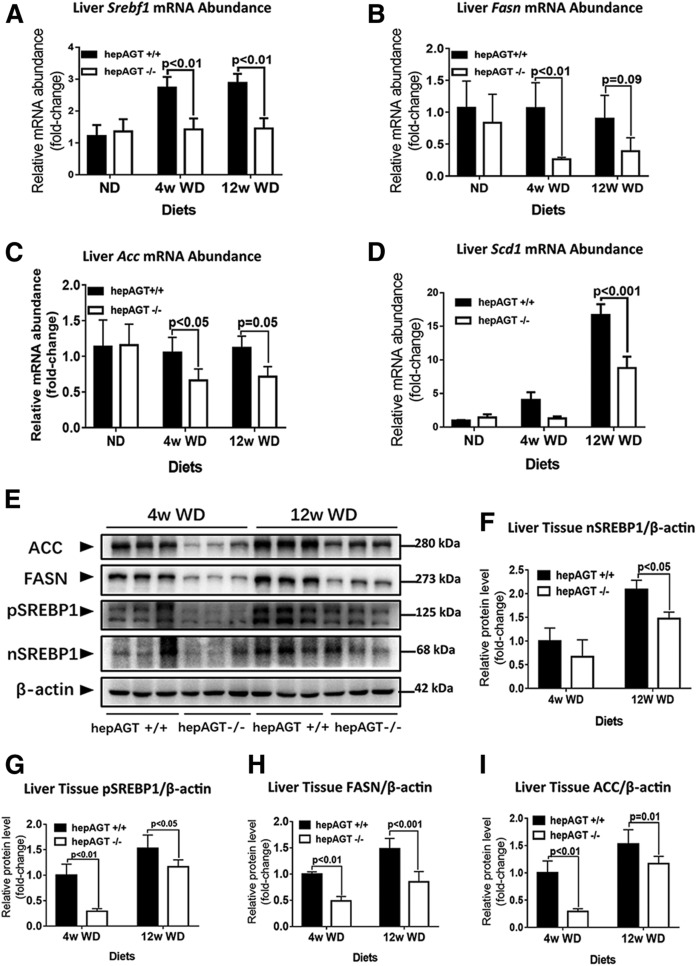
Loss of AGT in hepatocytes suppressed the SREBP-1c pathway. A: Liver *Srebf1* mRNA abundance identified by qPCR (N = 7–13 for each group). Comparison among groups by one-way ANOVA, Holm-Sidak post hoc test. B: Liver *Fasn* mRNA abundance identified by qPCR (N = 5–9 for each group). Comparison among groups by one-way ANOVA, Holm-Sidak post hoc test. C: Liver *Acc* mRNA abundance identified by qPCR (N = 5–9 for each group). Comparison among groups by one-way ANOVA, Holm-Sidak post hoc test. D: Liver *Scd1* mRNA abundance identified by qPCR (N = 5–9 for each group). Comparison among groups by one-way ANOVA, Holm-Sidak post hoc test. E–I: Representative images of liver tissue Western blotting (E) showed that n-SREBP1 (F), p-SREBP1 (G), FASN (H), and ACC (I) protein abundance was remarkably decreased in hepAGT^−/−^ mice compared with hepAGT^+/+^ mice when fed a Western diet (N = 3 for each group). Comparison among groups by two-way ANOVA, S-N-K post hoc test. ND, normal laboratory diet; WD, Western diet; p-SREBP1, precursor SREBP1; n-SREBP1, nuclear SREBP1.

**Fig. 5.**
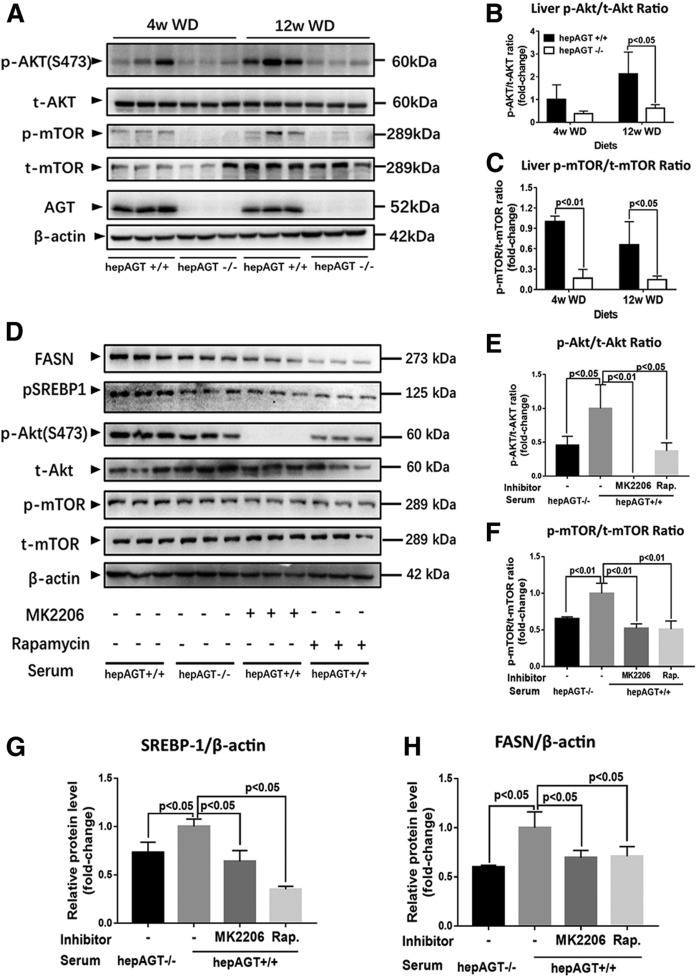
Hepatocyte-derived AGT induced the SREBP-1c pathway via Akt/mTOR signaling. A–C. Liver p-Akt and p-mTOR protein abundances were significantly lower in hepAGT^−/−^ mice compared with hepAGT^+/+^ mice fed a Western diet (N = 3 for each group). Comparison among groups by two-way ANOVA, S-N-K post hoc test. D–H: Representative Western blotting images (D) revealed that hepAGT^+/+^ mouse-derived serum treatment induced Akt/m-TOR phosphorylation (E, F) and upregulated SREBP1 (G) and FASN (H) protein abundance in mouse primary hepatocytes, and such effect could be abolished by MK2206, an Akt inhibitor, or rapamycin, a mTOR inhibitor. E–H: Primary hepatocytes were incubated with 0.5 mM PA in DMEM containing 10 nM insulin and 10% serum from either hepAGT^−/−^ mice or hepAGT^+/+^ mice for 6 h [MK2206 (10 nM, pretreated for 2 h); rapamycin (20 nM, pretreated for 30 min); N = 3 for each group]. Comparison among groups by one-way ANOVA, Holm-Sidak post hoc test. WD, Western diet; Rap., rapamycin.

Encoded by the sterol regulatory element-binding transcription factor 1 (*Srebf1*) gene, SREBP-1c is the major transcriptional factor that promotes hepatic lipogenesis; thereby, we next determined whether hepatic AGT deletion influenced SREBP-1c expression. When fed a Western diet, liver *Srebf1* expression was identified to be lower in hepAGT^−/−^ mice compared with the hepAGT^+/+^ mice (**Fig. 4A**). Furthermore, hepatic mRNA abundance of *Fasn*, *Acc*, and stearoyl-CoA desaturase 1 (*Scd1*), three critical downstream molecules of SREBP-1c involved in lipogenesis, was also significantly decreased in hepAGT^−/−^ mice versus hepAGT^+/+^ mice fed a Western diet (Fig. 4B–D). Accordingly, the protein levels of hepatic SREBP1, FASN, and ACC were reduced in the Western diet-fed hepAGT^−/−^ group relative to the hepAGT^+/+^ group (Fig. 4E–I). Such an effect could be mirrored in vitro when primary hepatocytes were stimulated with serum obtained from hepAGT^−/−^ and hepAGT^+/+^ mice, respectively, indicating that the absence of hepatocyte-derived AGT was associated with a remarkable suppression of the hepatic SREBP-1c pathway (**Fig. 5D**, G, H). In addition, we also detected an obvious reduction of expression of *Ppar-γ*, cell death-inducing DFF45-like effector (*Cide*)*-a*, and *Cide-c*, three important factors regulating lipid storage in hepAGT^−/−^ mice as compared with hepAGT^+/+^ mice after Western diet feeding (supplemental Fig. S5E–H).

### Hepatic AGT contributed to Akt/mTOR signaling-mediated SREBP1 activation in an Ang II-independent manner

We next attempted to define the mechanism of how hepatic AGT regulates hepatic lipogenesis, especially the link between hepatic AGT and the SREBP-1c pathway. Previous studies have verified that Akt phosphorylation activates the SREBP-1c pathway via mTOR complex 1 signaling, which provides the potential evidence that hepatic AGT may modulate SREBP-1c via the Akt/mTOR pathway (21–23). We then quantified the abundance of p-Akt and p-mTOR in liver tissue from hepAGT^+/+^ and hepAGT^−/−^ mice fed a Western diet. Surprisingly, hepAGT^−/−^ mice showed a remarkable reduction of p-Akt and p-mTOR abundance in hepatic tissue stimulated with Western diet (Fig. 5A–C, supplemental Fig. S6).

It is reported that hepatocyte-derived AGT contributes up to 90% of circulating AGT (14); thus, to further verify the alternation of the Akt/mTOR pathway associated with hepatic AGT deletion, primary hepatocytes were incubated with a culture medium containing 10% serum from either hepAGT^+/+^ or hepAGT^−/−^ mice, respectively (supplemental Fig. S7). Interestingly, hepAGT^+/+^ mouse-derived serum stimulation exacerbated hepatocyte steatosis in vitro (supplemental Fig. S8) and was associated with the Akt/mTOR pathway and subsequently promoted SREBP-1c expression, which could be diminished by treating with MK2206, an Akt inhibitor, and rapamycin, a mTOR inhibitor, respectively (Fig. 5).

**Fig. 6.**
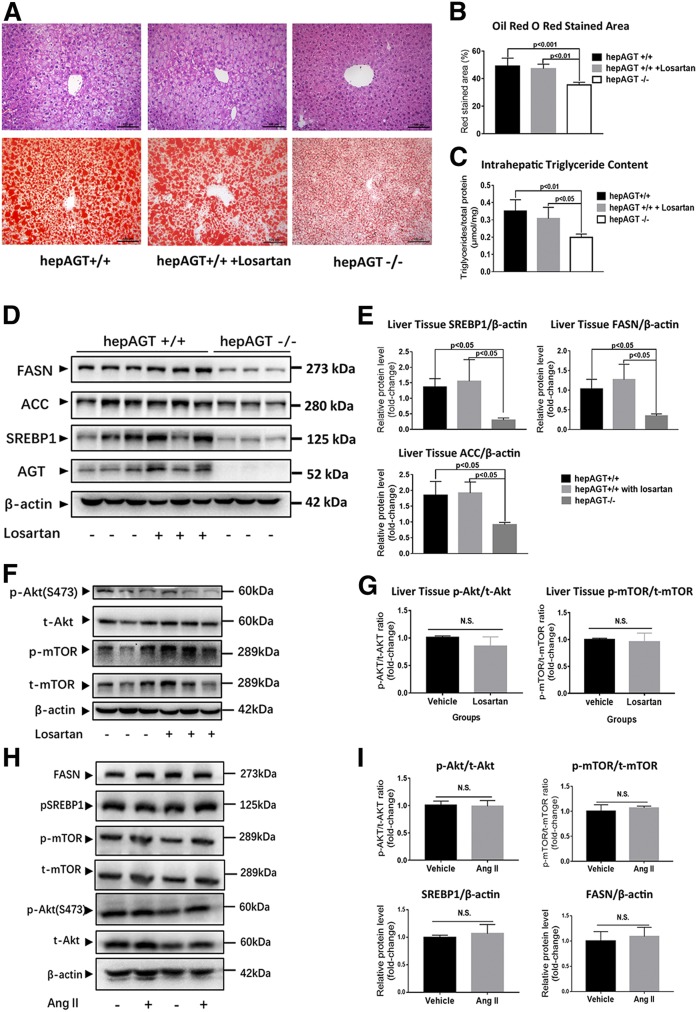
Hepatocyte-derived AGT promoted Akt/mTOR/SREBP-1c pathway activation in an Ang II-independent manner. A: The representative images of liver tissue section H&E (upper) and Oil Red O staining (lower) of hepAGT^+/+^ mice, hepAGT^−/−^ mice, and hepAGT^+/+^ mice that received losartan administration. All of these mice were fed a Western diet for 12 weeks. B: Quantification of the percentage of red-stained area in liver tissue section Oil Red O staining (N = 5 for each group). Comparison among groups by one-way ANOVA, Holm-Sidak post hoc test. C: Quantification of intrahepatic triglyceride contents of hepAGT^+/+^ mice, hepAGT^−/−^ mice, and hepAGT^+/+^ mice that received losartan administration (N = 5 for each group). Comparison among groups by one-way ANOVA, Holm-Sidak post hoc test. D, E: Western blotting revealed that losartan administration could not alter liver SREBP1, FASN, and ACC protein abundance in hepAGT^+/+^ mice fed a Western diet (N = 3 for each group). Comparison among groups by one-way ANOVA, Holm-Sidak post hoc test. F, G: Western blotting revealed that losartan administration could not alter liver Akt/mTOR signaling in hepAGT^+/+^ mice fed a Western diet (N = 3 for each group). Comparison between genotypes by Student’s *t*-test. H, I: In vitro Ang II (100 nM) stimulation exerted no significant effect on Akt/m-TOR signaling and SREBP1 and FASN protein abundance in mouse primary hepatocytes. Primary hepatocytes were incubated with 0.5 mM of palmitic acid in DMEM containing 10 nM of insulin with or without 100 nM of Ang II for 6 h (N = 3 for each group).

**Fig. 7.**
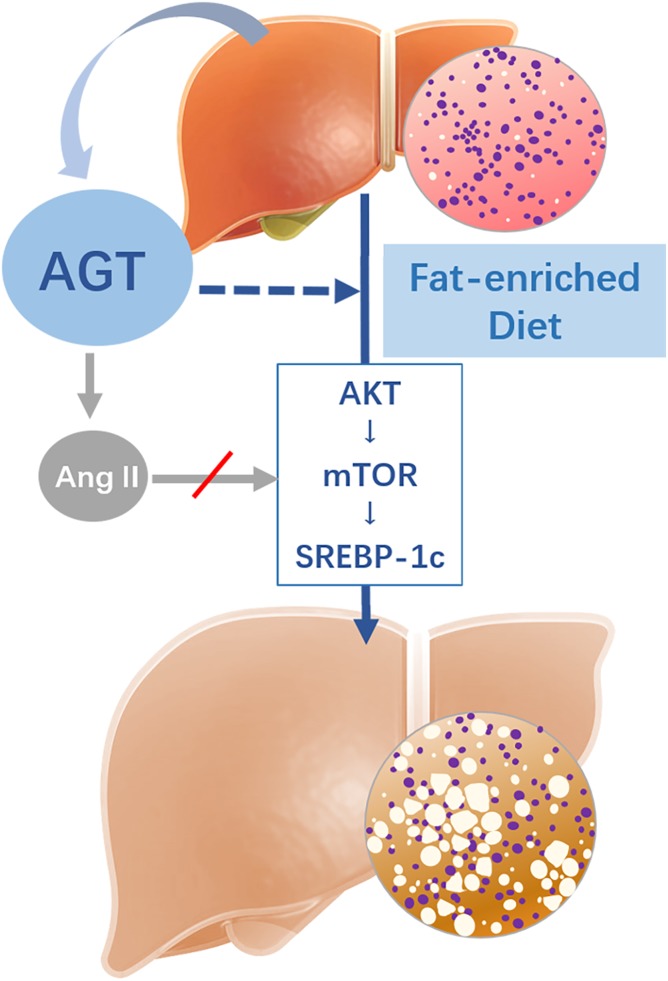
Potential mechanisms involved in hepatocyte-specific AGT contributing to Western diet-induced liver steatosis. Deletion of hepatocyte-derived AGT is associated with Akt/mTOR signaling alternation, subsequently reduces SREBP-1c expression, and ultimately alleviates abnormal lipid accumulation in liver tissue independent of Ang II signaling. The dashed arrow represents hepatic AGT involved in hepatic steatosis induced by Western diet. The gray arrow with a red slash represents an Ang II-independent manner.

Emerging studies have reported that Ang II can increase lipogenesis in adipocytes (24). Because AGT is the only precursor to generate all angiotensin peptides, including Ang II, we first hypothesized that AGT may promote the Akt/mTOR/SREBP-1c pathway in an Ang II-dependent manner. We then proceeded to block the effect of Ang II via an AT1R antagonist, losartan, and to determine whether mice treated with losartan exhibit a similar phenotype to hepAGT^−/−^ mice when fed a Western diet. Unexpectedly, hepAGT^+/+^ mice receiving losartan (10 mg/kg/day) displayed similar body weight gain, fat mass, and liver steatosis severity, as well as the abundance of p-Akt and p-mTOR compared with the mice treated with vehicle (**Fig. 6A**–G, supplemental Fig. S9). Conversely, we treated primary mouse hepatocytes with 100 nM Ang II in vitro, and no significant difference in protein abundance of p-Akt, p-mTOR, and SREBP1 was detected between vehicle- and Ang II-treated groups (Fig. 6H, I). Collectively, our results revealed that hepatic AGT might contribute to SREBP-1c upregulation via Akt/mTOR signaling in an Ang II-independent manner (**Fig. 7**).

## DISCUSSION

The role of the RAS in metabolic disorders remains controversial, which hinders efforts to manipulate RAS activity to achieve therapeutic efficacy. In the present study, we determined that hepatic AGT could promote Western diet-induced body weight gain and liver steatosis. Our results further mechanistically support that hepatocyte-derived AGT deletion is associated with inhibited Akt/mTOR phosphorylation, subsequently represses SREBP-1c expression, and ultimately alleviates lipid accumulation in liver tissue. Based on these findings, we highlight the key role of hepatocyte-specific AGT in the process of liver steatosis and may provide a novel approach for liver steatosis therapy.

### The pivotal role of hepatocyte-specific AGT on metabolic disorders

The global AGT-deficient mouse model was reported more than two decades ago (25, 26). Recently, a hepatocyte-specific AGT-deficient mouse model has been developed, which exerts a decrease of 90% in plasma AGT concentration (12). We investigated hepatocyte-specific AGT deficiency in mice compared with wild-type mice on a normal and a fat-enriched diet in the current study. The physiological differences of our mouse model versus global AGT KO mice are as follows: *1*) The global AGT-deficient mice have multiple health issues that may interfere with metabolic phenotypes. These mice not only have retarded growth, but also have a severe kidney defect (12). In contrast, hepAGT^−/−^ mice maintain normal growth and physiological functions. Therefore, we consider the hepAGT^−/−^ mouse model is more appropriate than whole-body AGT KO mice to study metabolic phenotypes. *2*) Metabolic phenotypes observed in hepAGT^−/−^ mice are somewhat different from those of global AGT KO mice. Global AGT KO mice gained less body weight than wild-type mice irrespective of normal diet or high-fat diet. However, hepAGT^−/−^ mice had comparable body weight gain when fed on normal diet but exhibited reduced body weight gain with improved insulin sensitivity when fed a Western diet. Additionally, when fed a fat-enriched diet, whole-body AGT KO mice showed less food intake, whereas hepAGT^−/−^ mice had comparable food intake as their hepAGT^+/+^ littermates. *3*) Global AGT deficiency displayed modest effects on liver weight, and its influence on liver steatosis was not reported. In contrast, hepatic AGT deficiency led to ablation of Western diet-induced liver steatosis, as evidenced by both liver triglyceride reduction and hepatic histologic changes. Collectively, the current study highlights the pivotal role of hepatocyte-specific AGT in metabolic disorders.

### The effect of hepatic AGT on lipid disposal

The intrahepatic lipid level is governed by lipid acquisition and disposal. So far, several major pathways have been identified to contribute to the modulation of liver lipid homeostasis. Liver acquires lipids through the uptake of circulating fatty acids and lipid synthesis. Conversely, lipids in hepatocytes are consumed via β-oxidation (especially in mitochondria) and exported as very low density lipoprotein particles (27). Therefore, in the current study, we identified the mechanisms linking hepatic AGT and liver steatosis via screening the above-mentioned pathways, which modulate liver lipid metabolism. Unexpectedly, the absence of hepatic AGT hardly affected the mitochondrial function of hepatocytes, especially on the oxidation of fatty acid. In addition, the concentration of plasma triglycerides, which is mainly contributed by the hepatic lipid export, was not altered in Western diet-fed hepAGT^−/−^ mice relative to hepAGT^+/+^ counterparts.

Emerging data have revealed that obesity and long-term high-fat diet feeding could impair the autophagosomal-lysosomal system (28). Actually, lipid droplets in the liver can be sequestrated by autophagosomes, which has been described and is termed lipophagy. Lipophagy has been reported to function on intracellular lipid turnover and energy homeostasis (29, 30). There is evidence to support that blockade of autophagy leads to retention of triglycerides and lipid droplets (31–33), while induction of hepatic autophagy alleviates metabolic stress and mitigates hepatic steatosis in ob/ob mice (34). Based on these findings, we further evaluated the effect of hepatic AGT deficiency on lipophagy. However, neither the abundance of autophagy-related proteins nor autophagic flux was altered in hepAGT^−/−^ mice fed a Western diet. Collectively, these negative findings suggest that hepatic AGT seems to exert a minor effect on hepatic lipid disposal.

### The effect of hepatic AGT on lipid acquisition

Despite the minor effect of hepatic AGT on lipid disposal, we identified the interesting phenotype in lipid acquisition induced by hepatic AGT. Fatty acid uptake and lipogenesis are the two major pathways that contribute to lipid acquisition. Fortunately, we identified a remarkably decreased abundance of SREBP-1c in hepatic tissue from hepAGT^−/−^ mice after Western diet feeding.

Encoded by the *Srebf1* gene, SREBP-1c is the predominantly expressed isoform of SREBPs in the liver. It is now recognized as a major transcriptional factor promoting hepatic de novo lipogenesis (35). The relationship between SREBP-1c and hepatic steatosis has been confirmed by several studies (35–37). Previous studies have indicated that the abundance of SREBP-1c is increased in NAFLD, and liver-specific SREBP-1c overexpression increased liver triglyceride content (35–39). In our study, the reduction of hepatic SREBP-1c abundance as well as attenuated liver steatosis was observed in hepAGT^−/−^ mice fed a Western diet. Furthermore, the expression of hepatic *Fasn, Acc*, and *Scd1*, three critical downstream targets of SREBP-1c, was also decreased in hepAGT^−/−^ mice fed a Western diet. Thus, our findings implicate that suppression of the SREBP-1c pathway may account for the alleviation of liver steatosis that resulted from hepatocyte-derived AGT deficiency.

Previous studies have illustrated that phosphorylation of Akt activates SREBP-1c through mTOR signaling (22, 23) or the Insig-2a pathway (21). In the current study, when fed a Western diet, the abundance of hepatic p-Akt and p-mTOR were reduced in hepAGT^−/−^ versus hepAGT^+/+^ mice in vivo. Interestingly, hepAGT^+/+^ mouse-derived serum stimulation upregulated Akt/mTOR phosphorylation and subsequently promoted SREBP-1c expression, which could be diminished by MK2206, an Akt inhibitor, or rapamycin, a mTOR inhibitor. Hence, we concluded that hepatic AGT deletion attenuated Western diet-induced liver steatosis and was associated with inhibition of Akt/mTOR/SREBP-1c signaling.

Emerging data have revealed that loss of hepatocyte-specific AGT in mice protects against Western diet-induced obesity independent of downstream components of the RAS system (14). Hence, the major concern was whether hepatocyte-specific AGT interfered with the Akt/mTOR/SREBP-1c pathway via Ang II signaling. The association between AGT and SREBP-1c-mediated lipogenesis has been described in several publications. For instance, overexpression of AGT has been reported to increase *Fasn* and *Srebf1* mRNA abundance in adipose tissues in vivo via the Ang II type 2 receptor (40). Other studies have demonstrated that AGT silencing downregulates expression of lipogenic genes, including *Srebf1* in 3T3-L1 adipocytes (24, 41). However, in our current study, hepAGT^+/+^ mice receiving losartan administration displayed a similar abundance of lipogenic proteins compared with the mice treated with vehicle. Conversely, in vitro Ang II stimulation could not alter activity of the SREBP-1c pathway. Based on these results, it seems that our findings are not supportive for hepatic AGT inducing the SREBP-1c pathway via Ang II-AT1R signaling.

### Limitations

Several limitations were identified in our study. First, the detailed mechanism underlying hepatic AGT and Akt/mTOR phosphorylation remains to be further identified. Second, in vivo functional assays for de novo fatty acid synthesis as well as very low density lipoprotein production were not performed in this study. Third, compared with hepAGT^+/+^ mice, Western diet-fed hepAGT^−/−^ mice also displayed extra-liver phenotypes including improved insulin sensitivity, less body weight gain, and less fat mass. The mechanistic link between hepatic AGT deficiency and extra-liver manifestation deserves further investigation.

In summary, our study illustrated that hepAGT^−/−^ mice exhibited attenuated liver steatosis with improved insulin sensitivity in response to Western diet. In response to fat-enriched diet, deletion of hepatocyte-derived AGT is associated with inhibited hepatic Akt/mTOR phosphorylation, downregulated SREBP-1c expression, and ultimately alleviated abnormal liver lipid accumulation independently of Ang II signaling.

## Supplementary Material

Supplemental Data
