# Supplementary material for: Angiotensinogen in hepatocytes contributes to Western diet-induced liver steatosis
Source: J Lipid Res. 2019 Oct 11;60(12):1983–95. doi: 10.1194/jlr.M093252 (PMC6889717; doi:10.1194/jlr.M093252)
Supplement: Supplemental Data [file supp_60_12_1983__index.html]

Angiotensinogen in Hepatocytes Contributes to Western Diet-induced Liver Steatosis — Angiotensinogen in hepatocytes contributes to Western diet-induced liver steatosis — Supplemental Data 

# Angiotensinogen in hepatocytes contributes to Western diet-induced liver steatosis

## Supplemental Data

- Supplemental information-method (.pdf, 404 KB) - supplemental methods
- Supplemental Figure S1 (.pdf, 249 KB) - Supplemental Figures-Figure S1
- Supplemental Figure S2 (.pdf, 454 KB) - Supplemental Figures-Figure S2
- Supplemental Figure S3 (.pdf, 352 KB) - Supplemental Figures-Figure S3
- Supplemental Figure S4 (.pdf, 269 KB) - Supplemental Figures-Figure S4
- Supplemental Figure S5 (.pdf, 639 KB) - Supplemental Figures-Figure S5
- Supplemental Figure S6 (.pdf, 299 KB) - Supplemental Figures-Figure S6
- Supplemental Figure S7 (.pdf, 240 KB) - Supplemental Figures-Figure S7
- Supplemental Figure S8 (.pdf, 346 KB) - Supplemental Figures-Figure S8
- Supplemental Figure S9 (.pdf, 440 KB) - Supplemental Figures-Figure S9
