# Supplementary material for: Angiotensinogen in hepatocytes contributes to Western diet-induced liver steatosis
Source: J Lipid Res. 2019 Oct 11;60(12):1983–95. doi: 10.1194/jlr.M093252 (PMC6889717; doi:10.1194/jlr.M093252)
Supplement: Supplemental Data [file 10.1194_M093252_jlr.M093252-1.pdf]

Supplemental Information

Angiotensinogen in Hepatocytes Contributes to Western Diet-induced Liver Steatosis

Xin-Ran Tao（陶昕然）, Jia-Bing Rong（戎佳炳）, Hong S. Lu（吕红）, Alan Daugherty, Peng Shi（史鹏）, Chang-Le Ke（柯昌乐）, Zhao-Cai Zhang（张召才）, Yin-Chuan Xu（徐银川）, Jian-An Wang（王建安）

From Department of Cardiology, Cardiovascular Key Laboratory of Zhejiang Province, Second Affiliated Hospital, Zhejiang University School of Medicine, Hangzhou, China (X.T., J.R., C.K., Y.X., and J.W.)

From Saha Cardiovascular Research Center University of Kentucky, Lexington (H.S.L., A.D.)

From Department of Pharmacology and Nutritional Sciences University of Kentucky, Lexington (H.S.L., A.D.)

From Department of Physiology University of Kentucky, Lexington (H.S.L., A.D.)

From Second Affiliated Hospital, Zhejiang University School of Medicine, Hangzhou, China (P.S.)

From Department of Intensive Care Unit, Second Affiliated Hospital, Zhejiang University School of Medicine, Hangzhou, China (Z.Z.)

Angiotensinogen in Hepatocytes Contributes to Western Diet-induced Liver Steatosis

Supplementary Methods

Genotyping of hepAGT +/+ and hepAGT-/- mice by PCR

Generation of hepatocyte-specific AGT knockout mice and structure of wild-type murine allele and floxed allele were described in previous study.(1) To identify mouse genotype, 1mm tail tissue was collected in clear tube. DNA was obtained by alkaline lysis method. Briefly, 75 µL 25 mM NaOH with 0.2 mM EDTA was added and heated at 98 °C for an hour. The lysates were neutralized with isovolumetric 40 mM Tris-Hcl solution (pH 5.5) and centrifuged at 2000 rpm for 2 minutes at room temperature. PCR was completed with GoTag Green Master Mix (M7123, Promega, USA). Each 20 µL PCR reaction system contains 10 µL GoTaq Green 2×Mix, 1 µL forward primer, 1 µl reverse primer, 3 µL DNA template, and was made up to 20 µL with PCR water.

Sequences of primers used for AGT floxed alleles were: LoxP F 5'-AAC CTT GTC TGG AGT GGG-3', LoxP R 5'- TCA GAG ATC CGT GGG AAC-3'.

The PCR program was listed below.

Program 1. 1 cycle - 94°C for 5 min

2. 35 cycles -94°C for 45 sec

56°C for 1 min

72°C for 1 min

3. 1 cycle - 72°C for 6 min

Sequences of primers used for AGT floxed alleles were:

IL2 (reference gene): F 5'-CTA GGC CAC AGA ATT GAA AGA TCT-3', R 5'-GTA GGT GGA AAT TCT AGC ATC ATC C-3';

Cre: F 5'-ACC TGA AGA TGT TCG CGA TT-3', R 5'-CGG CAT CAA CGT TTT CTT TT-3'.

Program 1. 1 cycle - 94℃ for 5 min

2. 35 cycles -94℃ for 45 sec

58.5℃ for 1 min

72℃ for 1 min

3. 1 cycle - 72℃ for 6 min

The mixture of PCR reaction was separated by 2.5% (w/v%) agarose gel. The results were presented in Figure S1.

The capacity of liver mitochondrial oxidation measurement

An Oxygraph-2k machine (O2k; OROBOROS Instruments, Innsbruck, Austria) was used to measure the oxidation capacity of isolated liver mitochondria. The isolated mitochondria in 100 μL preserving solution were added to the chamber with 2 mL MiR05 (respiration media containing 0.5 mM EGTA, 3mmol/L $MgCl_2$.6H2O, 60 mM potassium lactobionate, 20 mM taurine, 10 mM KH_2PO4, 20 mM HEPES, 110 mM sucrose, and 1g/L fatty acid-free BSA, pH 7.1).

The basal oxygen consumption rate (OCR) of isolated mitochondria was measured using 5 mM titration of pyruvate, 0.5 mM titration of malate, and 1.25 mM titration of ADP+Mg^{2+} as the substrates, and the maximum OCR was further measured after treatment with 0.05 μM titration of Carbonyl cyanide p-(trifluoro-methoxy) phenyl-hydrazone (FCCP) (C2920, Sigma, Saint Louis, MO, USA).(2)

Palmitic acid (PA) -BSA complex preparation

20%(w/v%) BSA solution was available by adding 5 g BSA powder to 25 mL 0.9% saline. pH of the solution was adjusted to 7.4 with 1 N NaOH. The solution is preserved on ice bath for further usage. Saponified PA (P0500, Sigma, Saint Louis, USA) was prepared as following steps. Briefly, to prepare 20 mM PA stock solution, 256.42 mg PA was added to a 50 mL tube containing 2 mL ethanol. Then 100 μL 5 N NaOH was added and mixed thoroughly. Finally, we evaporated the

aqueous ethanol solution to dry. PA-sodium solution was available by adding 15 mL 0.9% saline to the saponified PA prepared. Heat the solution to 75 ℃ for 30 minutes until PA is completely dissolved. The ice-cold BSA solution was warmed in 37 ℃ water bath, in which the PA-sodium solution was added. Adjust the final volume to 50 mL by adding 0.9% saline. Keep the solution at 37 ℃ for more than 30 minutes to allow PA binding to BSA. Filter the complex with 0.22 μm filter and aliquot, stored at -20 ℃.

Serum AGT concentration measurement

Mouse blood samples were collected via right ventricle after five hours of fasting and centrifuged at 400 g, 4 ℃ for 20 minutes to separate serum. Serum AGT concentration was measured using the Mouse Total Angiotensinogen Assay Kit (27413, IBL, Gunma, Japan) and followed the manufacturer's manual.

Quantitation of cellular Oil Red O staining

The method to quantify intracellular lipid-loading was described previously.(3) Briefly, hepatocytes were washed once with PBS after removing the supernatant, and then fixed with 4% formaldehyde for 15 minutes at room temperature. After removing the formaldehyde, Oil Red O working solution was filled into the culture plates to safely cover the plate bottom and incubated for 20 minutes at room temperature. After removing the staining solution, the plates were washed with distilled water for 5 times. Then the dye retained in the cells was eluted into isopropanol and O.D 490 nm was determined.

References

1. Lu, H., C. Wu, D. A. Howatt, A. Balakrishnan, J. J. Moorleghen, X. Chen, M. Zhao, M. J. Graham, A. E. Mullick, R. M. Crooke, D. L. Feldman, L. A. Cassis, C. W. Vander Kooi, and A. Daugherty. 2016. Angiotensinogen Exerts Effects Independent of Angiotensin II. *Arterioscler Thromb Vasc Biol*. **36**: 256-65.
2. Nan, J., H. Hu, Y. Sun, L. Zhu, Y. Wang, Z. Zhong, J. Zhao, N. Zhang, Y. Wang, Y. Wang, J. Ye, L. Zhang, X. Hu, W. Zhu, and J. Wang. 2017. TNFR2 Stimulation Promotes Mitochondrial Fusion via Stat3- and NF-kB-Dependent Activation of OPA1 Expression. *Circ Res*. **121**: 392-410.
3. Kraus, N. A., F. Ehebauer, B. Zapp, B. Rudolphi, B. J. Kraus, and D. Kraus. 2016. Quantitative assessment of adipocyte differentiation in cell culture. *Adipocyte*. **5**: 351-8.
