# Supplementary material for: Angiotensinogen in hepatocytes contributes to Western diet-induced liver steatosis
Source: J Lipid Res. 2019 Oct 11;60(12):1983–95. doi: 10.1194/jlr.M093252 (PMC6889717; doi:10.1194/jlr.M093252)
Supplement: Supplemental Data [file 10.1194_M093252_jlr.M093252-2.pdf]

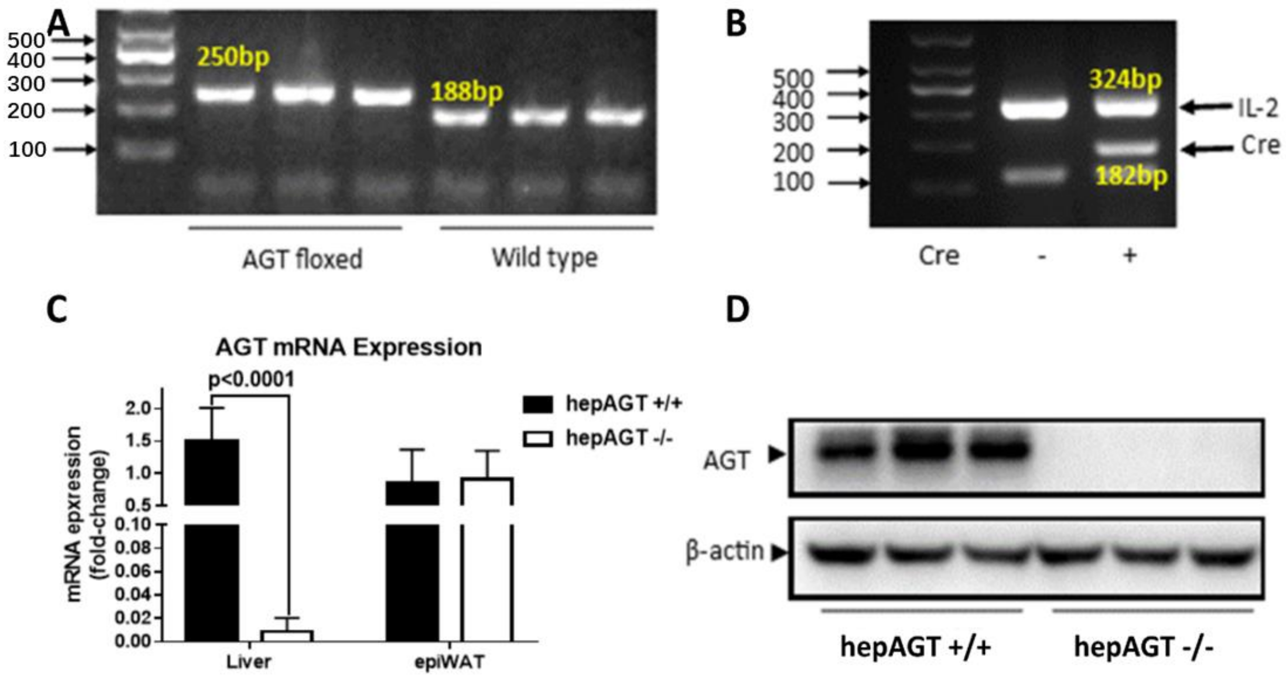

Figure S1 Generation of hepatocyte AGT specific deficient mice

A. Genotyping of the tail DNA of generated mice carrying the floxed allele and wild type allele.

B. Genotyping of the tail DNA of generated mice carrying the Alb-Cre.

C. AGT mRNA expression was decreased specificly in liver from hepAGT-/- mice, while AGT mRNA abundance in EpiWAT was similar between hepAGT+/+ and hepAGT-/- mice. N=4 to 9 for each group. Comparison between genotypes by Student's t-test.

D. Liver AGT protein abundance was significantly decreased in hepAGT-/- mice compared to hepAGT+/+ mice. N=3 for each group.

Cre: Cre recombinase. Epi-: Epidydimal.
