# Supplementary material for: Angiotensinogen in hepatocytes contributes to Western diet-induced liver steatosis
Source: J Lipid Res. 2019 Oct 11;60(12):1983–95. doi: 10.1194/jlr.M093252 (PMC6889717; doi:10.1194/jlr.M093252)
Supplement: Supplemental Data [file 10.1194_M093252_jlr.M093252-3.pdf]

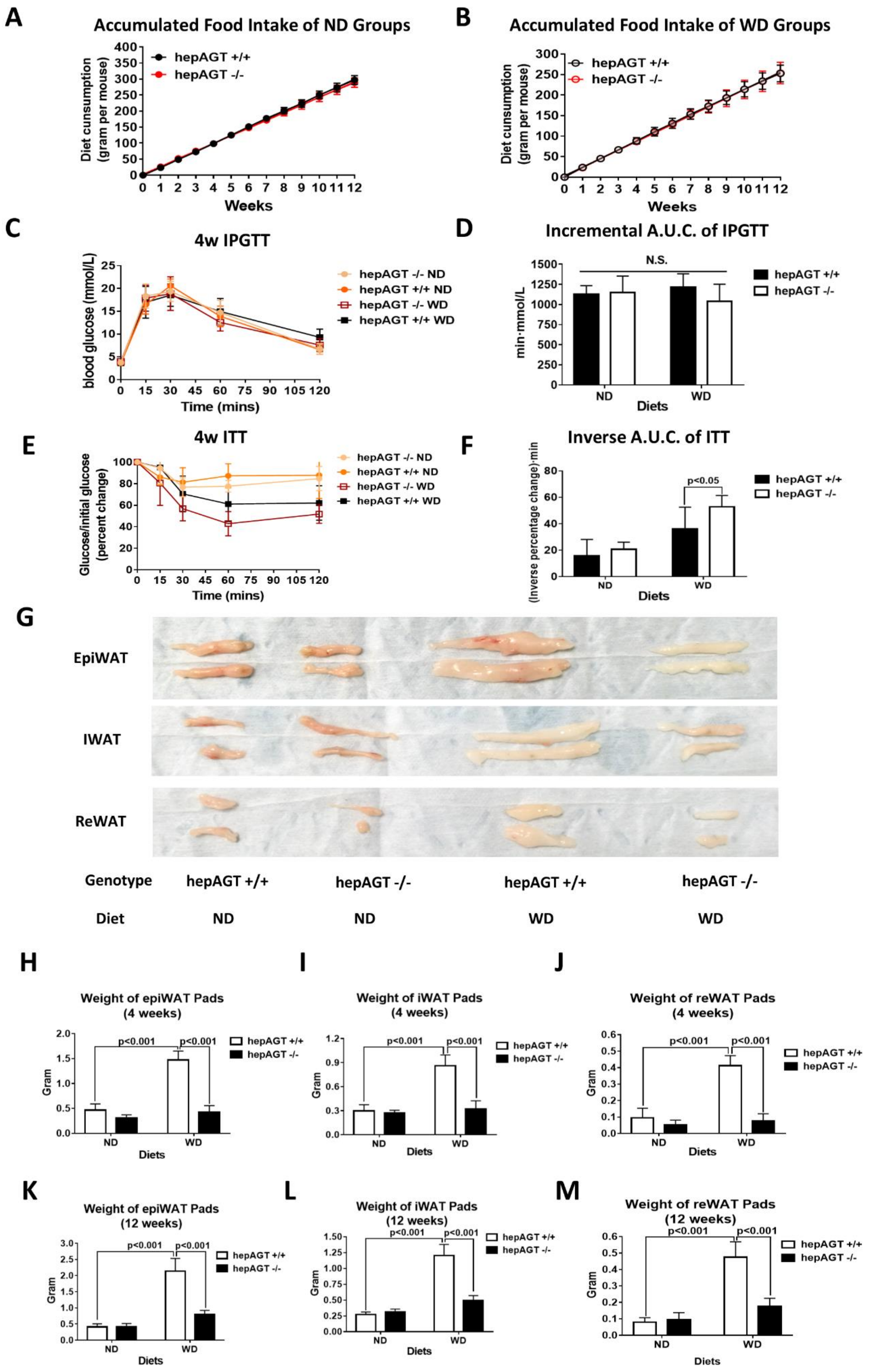

Figure S2 HepAGT-/- mice exhibited less fat mass when fed on western diet.

Figure S2 HepAGT-/- mice exhibited less fat mass when fed on western diet.

A. Accumulated diet consumption per mouse of hepAGT-/- and hepAGT+/+ group fed on normal laboratory diet. N=4 to 5 for each group. Comparison between genotypes by Two-Way Repeated Measurements on ANOVA.

B. Accumulated diet consumption per mouse of hepAGT-/- and hepAGT+/+ group fed on western diet. N=6 to 7 for each group. Comparison between genotypes by Two-Way Repeated Measurements on ANOVA.

C. Blood glucose level measured in IPGTT was similar between hepAGT-/- and hepAGT +/+ mice fed on normal laboratory diet and western diet respectively for 4 weeks. N=7 to 8 for each group. Comparison among groups by Two-Way ANOVA.

D. For IPGTT, incremental area under glucose curve was similar irrespective of genotype or diet. Comparison among groups by Two-Way ANOVA.

E. Blood glucose level measured in ITT was similar between hepAGT-/- and hepAGT +/+ mice fed on normal laboratory diet and western diet respectively for 4 weeks. N=6 to 8 for each group.

F. For ITT, inverse area under glucose curve was significantly larger in hepAGT-/- mice group than that of hepAGT +/+ littermates after 4 weeks of western diet feeding. Comparison among groups by Two-Way ANOVA.

G. Typical images of fat pads of EpiWAT, IWAT and ReWAT from hepAGT-/- and hepAGT +/+ mice fed on normal laboratory diet or western diet for 12 weeks.

H-J. After 4 weeks of western diet feeding, weights of EpiWAT (H), IWAT (I), and ReWAT (J) were remarkably decreased in hepAGT-/- mice compared to hepAGT+/+ mice. Comparison among groups by Two-Way ANOVA, S-N-K post hoc test.

K-M. After 12 weeks of western diet feeding, weight of EpiWAT (K), IWAT (L), and ReWAT (M) was remarkably decreased in hepAGT-/- mice compared to hepAGT+/+ mice. Comparison among groups by two-way ANOVA, S-N-K post hoc test.

Epi-: Epidydimal. I-: inguinal. Re-: Retroperitoneal. WAT: White adipose tissue.
