# Supplementary material for: Angiotensinogen in hepatocytes contributes to Western diet-induced liver steatosis
Source: J Lipid Res. 2019 Oct 11;60(12):1983–95. doi: 10.1194/jlr.M093252 (PMC6889717; doi:10.1194/jlr.M093252)
Supplement: Supplemental Data [file 10.1194_M093252_jlr.M093252-4.pdf]

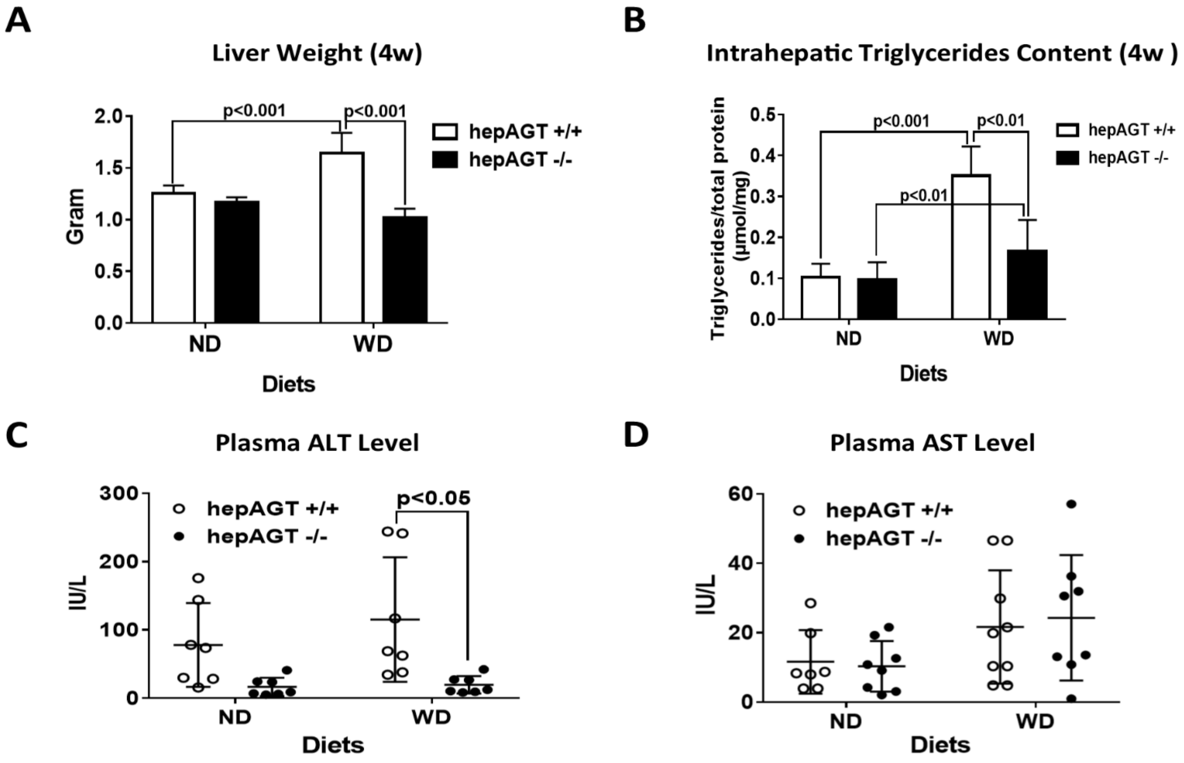

Figure S3 HepAGT-/- mice exhibited less liver weight, decreased intrahepatic triglyceride content and attenuated hepatic injury in response to western diet.

A. Hepatic AGT deletion ameliorated western diet induced liver weight gain. (1.6 ± 0.2 g for hepAGT +/+ vs 1.0 ± 0.1 g for hepAGT-/-, p<0.001). N=4 to 7 for each group. Comparison among groups by two-way ANOVA, S-N-K post hoc test. Comparison among groups by two-way ANOVA, S-N-K post hoc test.

B. Intrahepatic triglyceride contents were lower in hepAGT-/- mice after 4 weeks of western diet feeding. (ND: 0.096±0.043 μmol/mg for hepAGT-/- vs 0.102±0.033 μmol/mg for hepAGT +/+. WD: 0.167±0.008 μmol/mg for hepAGT -/- vs 0.35±0.071 μmol/mg for hepAGT +/+, p<0.01). N=6 to 9 for each group. Comparison among groups by two-way ANOVA, S-N-K post hoc test.

C. HepAGT-/- mice exhibited lower plasma ALT level compared to hepAGT +/+ mice fed on a western diet. (69.0 (34.3, 244.2) IU/L for hepAGT +/+ vs 12.5 (8.5, 42.0) IU/L for hepAGT -/- in western diet cohort, p<0.05). N=7 to 9 for each group. Comparison among groups by ANOVA on Ranks, Tukey post hoc test.

D. Plasma AST level was slightly elevated in response to western diet and was similar between hepAGT-/- mice and hepAGT +/+ mice when fed on western diet. N=7 to 9 for each group. Comparison among groups by ANOVA on Ranks.

ND: Normal laboratory diet. WD: Western diet. ITT: Insulin tolerance test. IPGTT: Intraperitoneal glucose tolerance test. A.U.C: Area under curve.
