# Supplementary material for: Angiotensinogen in hepatocytes contributes to Western diet-induced liver steatosis
Source: J Lipid Res. 2019 Oct 11;60(12):1983–95. doi: 10.1194/jlr.M093252 (PMC6889717; doi:10.1194/jlr.M093252)
Supplement: Supplemental Data [file 10.1194_M093252_jlr.M093252-5.pdf]

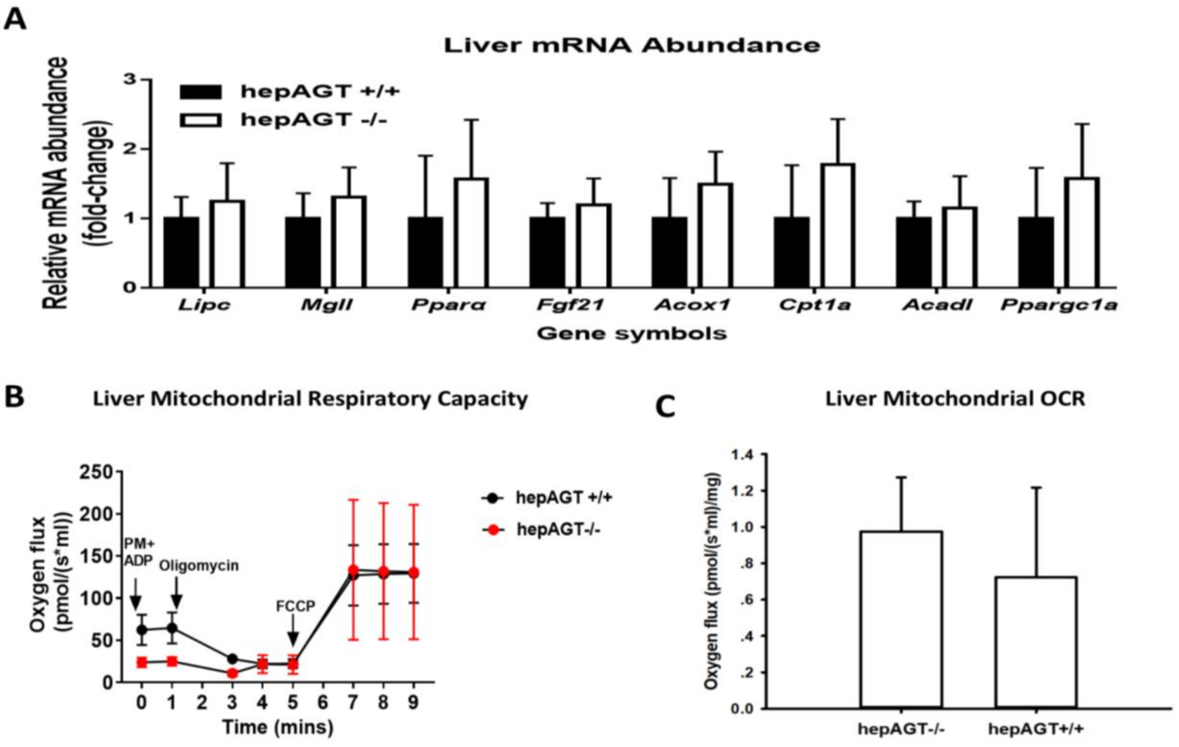


**Figure S4 Hepatocyte-specific AGT deletion exerted minor effect on expressions of genes involved in lipolysis, fatty acid uptake and mitochondrial oxidation capacity.**

A. mRNA abundance of genes involved in liver lipolysis, fatty acid uptake was similar between hepAGT-/- mice and hepAGT+/+ mice fed on normal laboratory diet. N=3 to 9 for each group. Comparison between genotypes by Student's t-test.

B. The oxygen flux curves in respiratory assays of liver mitochondria isolated from hepAGT+/+ mice and hepAGT-/- mice fed on western diet, respectively. Comparison between genotypes by Student's t-test.

C. The oxygen consumption rate was similar between hepAGT-/- mice and hepAGT +/+ mice fed on western diet using a high-resolution respirometry. N=3 for each group. Comparison between genotypes by Student's t-test.

P: Pyruvic acid sodium salt. M: L-Malic acid. ADP: Adenosine 5'-diphosphate. FCCP: Carbonyl cyanide p-(trifluoro-methoxy) phenyl-hydrazone.
