# Supplementary material for: Angiotensinogen in hepatocytes contributes to Western diet-induced liver steatosis
Source: J Lipid Res. 2019 Oct 11;60(12):1983–95. doi: 10.1194/jlr.M093252 (PMC6889717; doi:10.1194/jlr.M093252)
Supplement: Supplemental Data [file 10.1194_M093252_jlr.M093252-6.pdf]

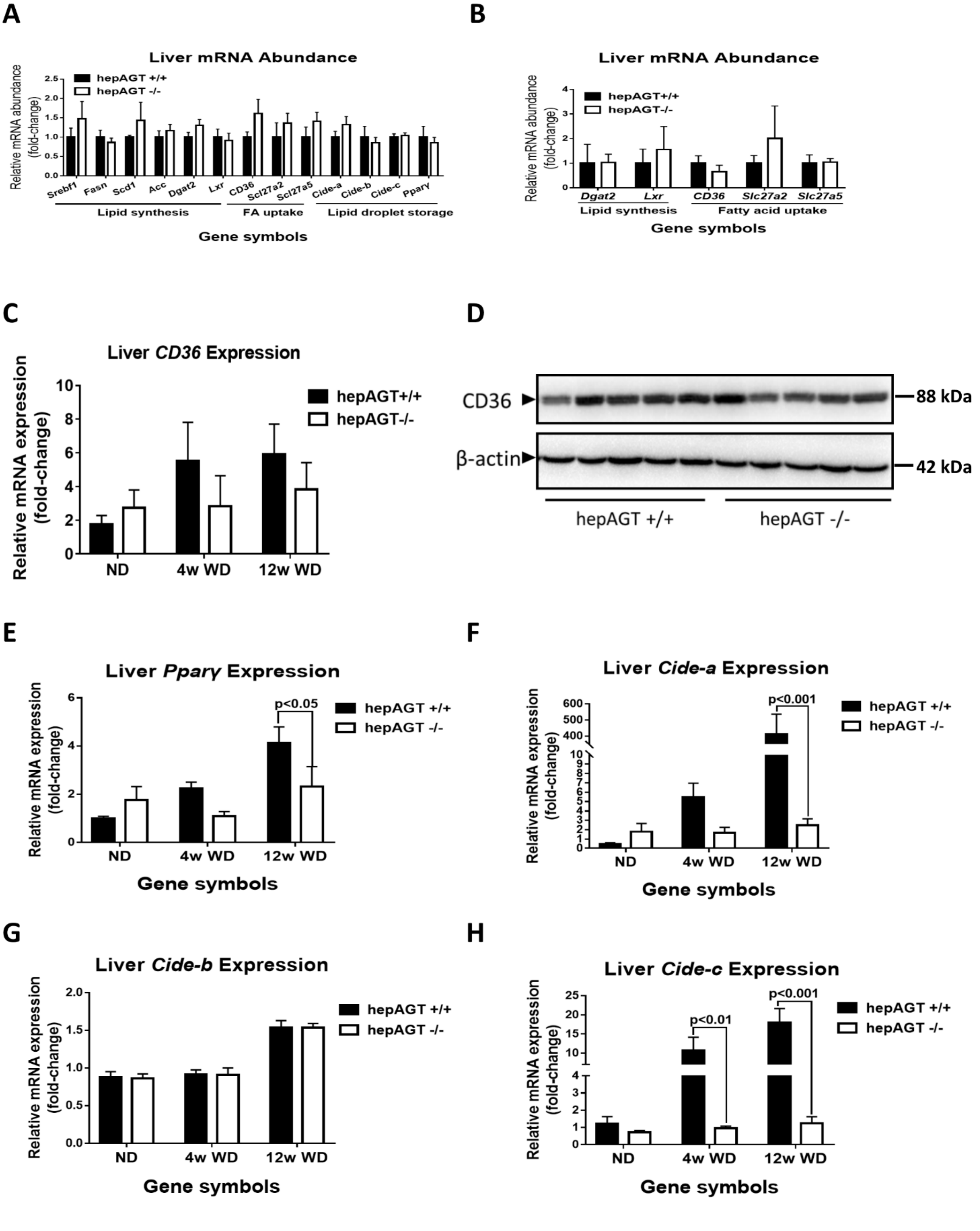

Figure S5 Liver mRNA abundance of fatty acid uptake related genes were barely affected by hepatic AGT deficiency, while hepatic mRNA abundance of genes promoting lipid droplet storage was reduced in hepAGT-/- mice after western diet feeding.

Figure S5 Liver mRNA abundance of fatty acid uptake related genes were barely affected by hepatic AGT deficiency, while hepatic mRNA abundance of genes promoting lipid droplet storage was reduced in hepAGT-/- mice after western diet feeding.

A. Liver mRNA abundance of genes involved in liver lipid synthesis, fatty acid uptake, and lipid storage was similar between hepAGT-/- mice and hepAGT+/+ mice fed on normal laboratory diet. N=3 to 9 for each group. Comparison between genotypes by Student's t-test.

B. Hepatic mRNA of genes involved in liver fatty acid uptake was not affected by hepatocyte-specific AGT deficiency in response to western diet. N=5 to 7 for each group. Comparison between genotypes by Student's t-test.

C. Hepatic mRNA abundance of CD36 was not affected by hepatocyte-specific AGT deficiency in response to western diet. N=5 to 7 for each group. Comparison among groups by One-Way AVONA, Holm-Sidak post hoc test.

D. Hepatic protein abundance of CD36 was not affected by hepatocyte-specific AGT deficiency in response to western diet. N=5 for each group.

E. Hepatic AGT deletion suppressed western diet-induced hepatic *Pparγ* expression. N=5 to 9 for each group. Comparison among groups by One-Way AVONA, Holm-Sidak post hoc test.

F. Hepatic AGT deletion suppressed western diet-induced hepatic *Cide-a* expression. N=5 to 9 for each group. Comparison among groups by One-Way AVONA, Holm-Sidak post hoc test.

G. Liver *Cide-b* mRNA abundance was not affected by absence of hepatocyte-derived AGT. N=5 to 9 for each group. Comparison among groups by One-Way AVONA, Holm-Sidak post hoc test.

H. Hepatic AGT deletion suppressed western diet-induced hepatic *Cide-c* expression. N=5 to 9 for each group. Comparison among groups by One-Way AVONA, Holm-Sidak post hoc test.

Pparγ: Peroxisome proliferator-activated receptor γ. *Cide*: Cell death-inducing DFF45-like effectors.
