# Supplementary material for: Angiotensinogen in hepatocytes contributes to Western diet-induced liver steatosis
Source: J Lipid Res. 2019 Oct 11;60(12):1983–95. doi: 10.1194/jlr.M093252 (PMC6889717; doi:10.1194/jlr.M093252)
Supplement: Supplemental Data [file 10.1194_M093252_jlr.M093252-7.pdf]

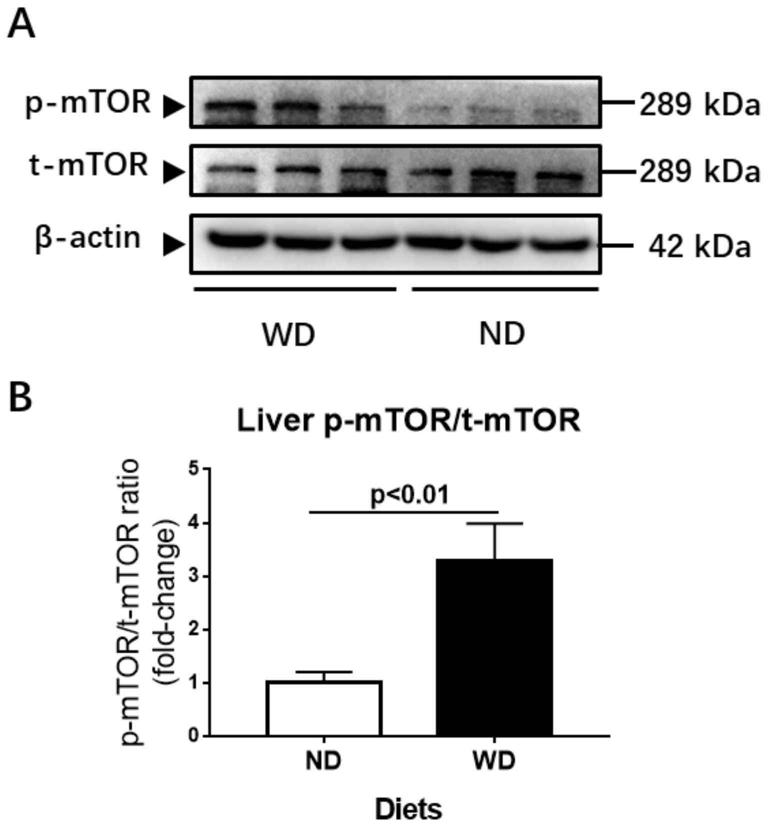

Figure S6 Western diet induced an elevation of hepatic p-mTOR protein abundance in hepAGT+/+ mice.

A. Representative western blotting images revealed that hepatic p-mTOR protein abundance was induced by western diet feeding *in vivo*.

B. Quantification of hepatic p-mTOR/t-mTOR ratio. N=3 for each group. Comparison between genotypes by Student's t-test.
