# Supplementary material for: Angiotensinogen in hepatocytes contributes to Western diet-induced liver steatosis
Source: J Lipid Res. 2019 Oct 11;60(12):1983–95. doi: 10.1194/jlr.M093252 (PMC6889717; doi:10.1194/jlr.M093252)
Supplement: Supplemental Data [file 10.1194_M093252_jlr.M093252-8.pdf]

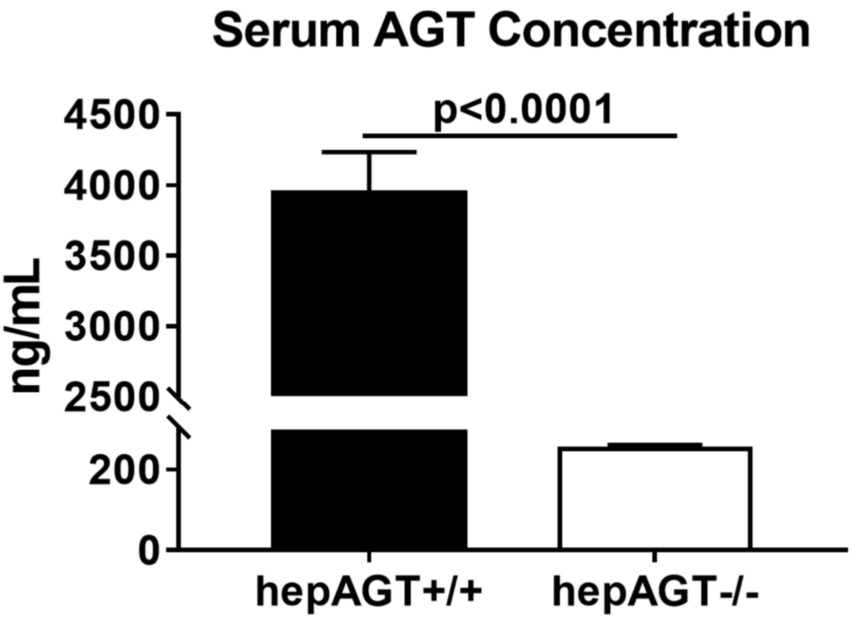

Figure S7 Serum AGT concentration was lower in hepAGT-/- mice than in hepAGT+/+ mice.
The concentrations of AGT in serum samples added to hepatocyte culture medium were measured by ELISA. N=5 for each group. Comparison between genotypes by Student’s t-test.
