# Supplementary material for: Angiotensinogen in hepatocytes contributes to Western diet-induced liver steatosis
Source: J Lipid Res. 2019 Oct 11;60(12):1983–95. doi: 10.1194/jlr.M093252 (PMC6889717; doi:10.1194/jlr.M093252)
Supplement: Supplemental Data [file 10.1194_M093252_jlr.M093252-9.pdf]

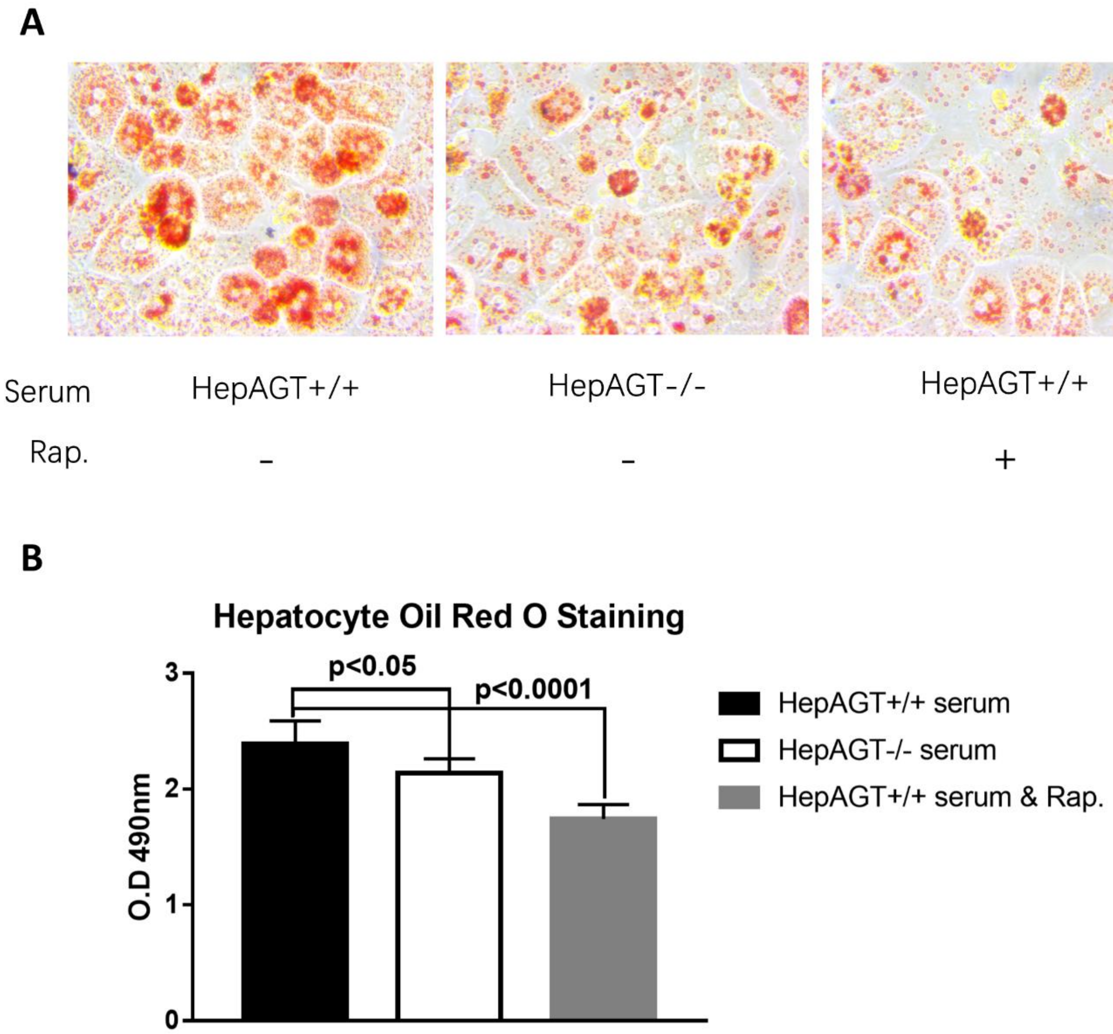


**Figure S8 Lipid accumulation in hepatocytes induced by serum obtained from hepAGT+/+ mice was ameliorated by rapamycin treatment.**

A. Representative images of Oil Red O stained hepatocytes treated with 10% hepAGT-/-serum, 10% hepAGT+/+ serum, and 10% hepAGT+/+ serum with rapamycin, respectively. (400×) (Primary hepatocytes were incubated with 0.5 mM PA in DMEM containing 10 nM insulin and 10% serum from either hepAGT-/- mice or hepAGT+/+ mice, respectively for six hours. Rapamycin: 20 nM, pretreated for 30 minutes.)

B. Quantitation of hepatocyte Oil Red O staining. N=6 for each group. Comparison among groups by One-Way AVONA, Holm-Sidak post hoc test.
