# Supplementary material for: Angiotensinogen in hepatocytes contributes to Western diet-induced liver steatosis
Source: J Lipid Res. 2019 Oct 11;60(12):1983–95. doi: 10.1194/jlr.M093252 (PMC6889717; doi:10.1194/jlr.M093252)
Supplement: Supplemental Data [file 10.1194_M093252_jlr.M093252-10.pdf]

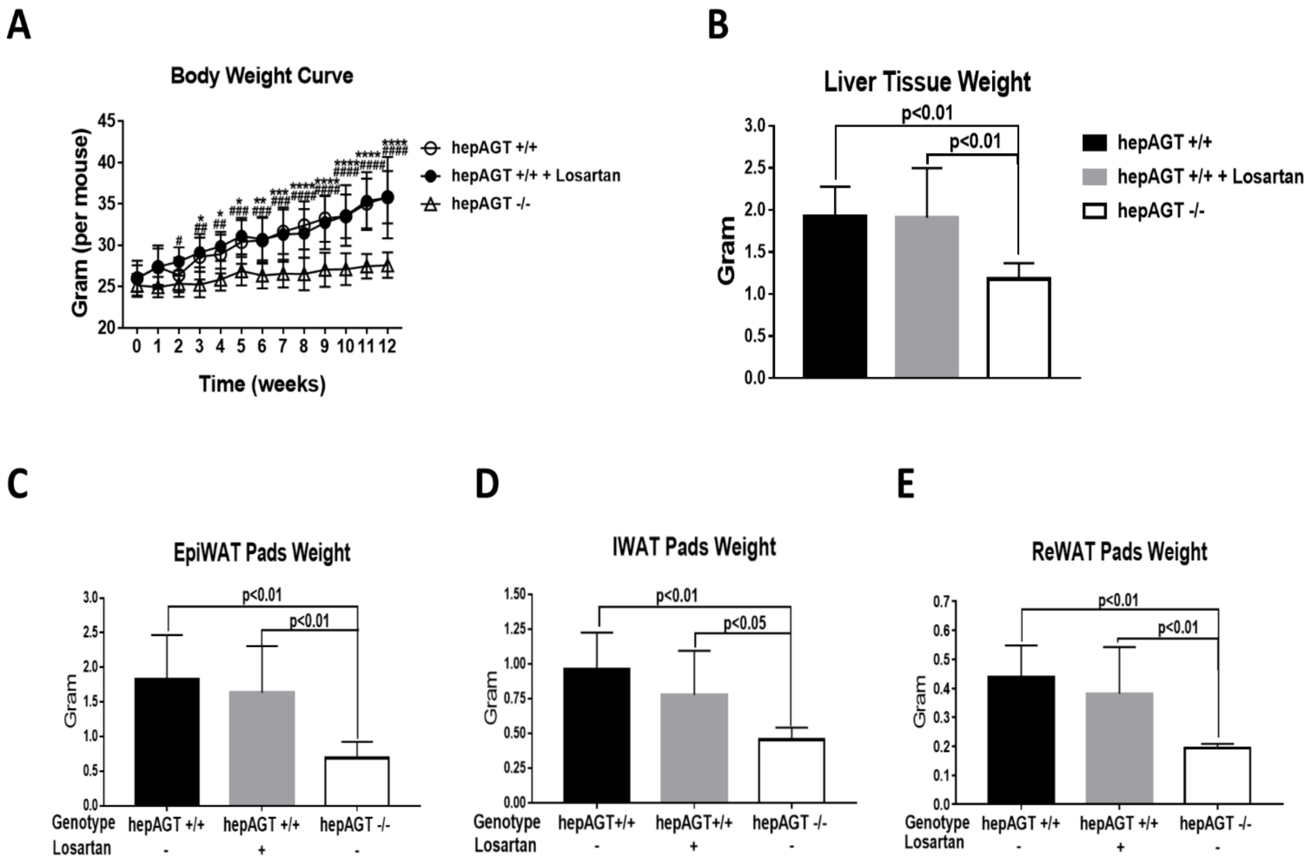

Figure S9 Losartan administration could not affect western diet induced whole body, liver and adipose weight gain.

A. HepAGT+/+ mice treated with 10 mg/Kg/day losartan exhibited similar body weight gain compared to hepAGT+/+ mice received vehicle in response to western diet. N=6 to 9 for each group. Comparison among groups by One-Way AVONA, Holm-Sidak post hoc test.

B. HepAGT+/+ mice treated with 10 mg/kg/day losartan exhibited similar liver weight compared to hepAGT+/+ mice received vehicle in response to western diet. N=6 to 9 for each group. Comparison among groups by One-Way AVONA, Holm-Sidak post hoc test.

C-E. Weight of fat pads of EpiWAT (C), IWAT(D), and ReWAT (E) showed that *in vivo* losartan treatment had no effect on western diet-induced fat gain in hepAGT+/+ mice. N=6 to 9 for each group. Comparison among groups by One-Way AVONA, Holm-Sidak post hoc test.
